# Targeting Myadm to Intervene Pulmonary Hypertension on Rats Before Pregnancy Alleviates the Effect on Their Offspring’s Cardiac-Cerebral Systems

**DOI:** 10.3389/fphar.2021.791370

**Published:** 2022-01-18

**Authors:** Jingrong Wang, Zirui Zhang, Cui Liang, Tingting Lv, Haoying Yu, Shuyue Ren, Peirong Lin, Guanhua Du, Lan Sun

**Affiliations:** ^1^ Institute of Materia Medica, Chinese Academy of Medical Science and Peking Union Medical College, Beijing, China; ^2^ The State Key Laboratory of Bioactive Substance and Function of Natural Medicines, Beijing, China; ^3^ Department of Anesthesiology, Beijing Anzhen Hospital, Capital Medical University, and Beijing Institute of Heart, Lung, and Blood Vessel Diseases, Beijing, China

**Keywords:** pulmonary hypertension, pregnancy, low birth weight, cardiac-cerebral system, Myadm, development

## Abstract

Pregnancy with pulmonary hypertension (PH) seriously threatens the life and safety of mothers and infants. Here, the long-term effect of maternal PH on the postpartum growth of rat offspring was focused for the first time, as well as explored the role of Myadm in PH rats before pregnancy based upon the previous findings. Patients with PH are prone to hypoxemia, leading to insufficient placental structure and function, which affects the organ function of fetuses, followed by evidence that differently expressed genes (DEGs) existed in the heart of maternal PH newborn rats and enriched in pathways related to cardiac and nerve development on human infants with similar birth outcome: low birth weight (LBW). LBW was one of the possible birth outcomes of pregnancy with PH, especially severe PH, accompanied by evidence that offspring derived from mothers with PH presented lower birth weights and slower growth rates than those derived from normal control mothers in a rat model. Besides, maternal PH rat offspring showed cardiac remodeling and a significant elevation of the expression levels of hypoxia- and inflammation-related markers in the cerebral cortex at both 10 and 14 weeks of age, respectively. What is more, the previous studies found that the overexpression of Myadm could result in the remodeling of the pulmonary artery. And targeting Myadm to intervene PH before pregnancy could alleviate sustained low weight growth in maternal PH rat offspring, and the pathological changes of the cardiac–cerebral system caused by maternal PH, including enlarged right heart cavity, loss of cardiomyocytes, abnormal heart index, as well as cerebral cortex hypoxia and the inflammatory state as they grew up to a certain extent. The findings show the pathological significance of maternal PH on offspring growth and the cardiac–cerebral development in a rat model, as well as point out the potential treatment target, which may provide a further reference for pregnancy outcomes in women with PH and healthy development of offspring to some extent.

## Introduction

Pulmonary hypertension (PH) is a life-threatening, complex group of disorders that result from different pathophysiologic mechanisms but all clinically defined as mean pulmonary arterial pressure (mPAP) greater than 25 mmHg (1 mmHg = 0.133 kPa) at rest by the right heart catheterization (RHC), commonly including pulmonary arterial hypertension (PAH), chronic thromboembolic pulmonary hypertension (CTEPH), and PH due to left heart and lung diseases (Galie et al., 2016; Cai et al., 2018). Currently, an increasing number of studies have noted the sex differences in PH (Gabler et al., 2012; Liu et al., 2017). In particular, epidemiological data show a higher incidence rate of PAH in women (Hoeper et al., 2016). In addition, women who are diagnosed with PH are usually of child-bearing age (Sahni et al., 2015; Hill et al., 2018). Unfortunately, pregnancy is not recommended in patients with PH because of intolerable, hemodynamic, anatomic, and biochemical changes (Sun et al., 2018), and the high mortality rates up to approximately 30–56% and 11–28% of mothers and infants, respectively (Weiss et al., 1998; Olsson and Channick, 2016). Thus, timely termination of pregnancy seems to be an effective way to improve cardiac function and pregnancy outcomes, especially for patients with severe PH (Monagle et al., 2015).

Numerous clinical studies have paid much attention to the treatment and management of mothers, as well as fetal birth outcomes. As was reported, the adverse birth outcomes of pregnancy with PH included premature delivery, low birth weight (LBW), small for gestational age (SGA), intrauterine growth restriction (IUGR), and even death (Duan et al., 2016; Sliwa et al., 2016; Keepanasseril et al., 2019; Jha et al., 2020). Inspiringly, it has been noted that pregnancy outcomes are improved due to multidisciplinary cooperation (Rezk and Gamal 2016). A previous study suggested that pregnancy might not be absolutely contraindicated in women with moderate PH (Lai et al., 2021). However, there is still an absolute risk in pregnancy with PH involving maternal and neonatal complications (Balieva et al., 2021). However, while fetal birth outcomes have been widely concerned nowadays, whether maternal PH surviving offspring show growth differences in childhood, adolescence, adulthood, or senescence is still unknown due to the difficulty of long-term follow-up. For surviving infants, does maternal PH influence the growth and development of their offspring? If so, how long will the impact exist? Would the impact of adverse intrauterine environments caused by maternal PH continue to affect the postnatal growth of offspring? Considering the above knowledge gap, it was focused on providing relevant data for further guidance on timely intervention to ensure the healthy growth of offspring.

In the present study, public databases and gene expression profile analysis combined with preclinical experiments were used to explore the influence of maternal PH on offspring’s development after birth. The gene expression profiles of PH patients, the fetus with similar birth outcomes, and maternal PH rat offspring were analyzed. Then, a Sprague–Dawley (SD) rat model of pregnancy with monocrotaline (MCT)-induced PH was established. According to the results, the pathological changes of their offspring were observed in the growth state, and cardiac and cerebral systems, and explored the related mechanisms. In addition, the pathological mechanism of PH has been widely studied by using Omics recently. Myeloid-associated differentiation marker (Myadm), a novel hematopoietic-associated marker composed of 322 amino acids broadly expressed in different tissues and cells (Yagil et al., 2005; Sun et al., 2020; Bai et al., 2021), was found to significantly associate with long-term changes in blood pressure (Zeller et al., 2017). It was found that the expression of Myadm was increased in the neointima, suggesting that Myadm is a potential target of vascular remodeling (Sun et al., 2016). Besides, the previous studies found a new regulatory model of miR-182-3p/Myadm/KLF4/p21 axis in PH vascular remodeling (Sun et al., 2020). Based on the previous findings, Myadm was targeted to intervene PH before pregnancy and explored its influence on the development of maternal PH offspring.

## Materials and Methods

### Analysis of Gene Expression Profiles of Patients With PH Based Upon the GEO Database

“Pulmonary hypertension” was searched in the Gene Expression Omnibus (GEO) database. Microarray datasets (GSE113439 platform: GPL6244 platform, Affymetrix Human Gene 1.0 ST Array [transcript (gene) version]) were downloaded, which enrolled 15 fresh frozen lung samples from patients with PH and 11 normal control tissue flanking lung cancer resections. GEO2R online software was used to screen the differentially expressed genes (DEGs). R language software (version 4.0.0) was applied to construct volcano maps. Then, the Database for Annotation, Visualization, and Integrated Discovery (DAVID, version 6.8) was used to perform functional and pathway enrichment analyses of screened DEGs. The R software package was used to visualize the results of Genome Ontology (GO) and Kyoto Encyclopedia of Genes and Genomes (KEGG) pathway enrichment analyses. In addition, the Search Tool for the Retrieval of Interacting Genes (STRING, version 11.0) database was used to construct a protein–protein interaction (PPI) network of DEGs, and the significant module in the PPI network was calculated using Cytoscape software (version 3.7.2).

### Analysis of the Correlation Between Human Maternal PH Severity and Fetal Birth Weight

“Pregnancy with pulmonary hypertension,” “birth outcomes,” and “low birth weight” were searched in PubMed and Clinical Trials. Relevant pieces of English literatures were obtained, and available cases of pregnancy with PH were collected. In addition, because detailed individual case reports usually contain different interventions and pay more attention to the treatments and management of mothers, after fully considering the statistical problems due to the differences in individual case reports, different series of cases from different single centers, including information on both maternal pulmonary artery pressure and neonatal birth weight, were selected in the present study. Patients were divided into the mild group (30–50 mmHg), moderate group (50–70 mmHg), and severe group (>70 mmHg) according to their systolic pulmonary artery pressure (SPAP). Their fetal birth weights were extracted correspondingly and were included in correlation analysis.

### Analysis of Gene Expression Profiles Related to Human Infants With LBW Based Upon the GEO Database

“Low birth weight,” “small for gestational age,” and “intrauterine growth restriction” were searched in the GEO database. Gene expression profiling datasets (GSE24818 platform: GPL6480 Agilent-014850 Whole Human Genome Microarray 4x44K G4112F) were acquired, which contained umbilical cord tissue from 18 infants with LBW and 22 infants with normal birth weight (NBW). GEO2R online software was used to analyze datasets and find the DEGs (version 4.0.0). R language software was applied to construct volcano maps and heat maps. Then, the DAVID (version 6.8) was chosen to perform functional and pathway enrichment analyses of screened DEGs. In addition, the STRING database (version 11.0) and Cytoscape software (version 3.7.2) were used to construct a PPI network of DEGs.

### Establishment of Maternal PH in Rats

Sprague–Dawley (SD) rats (6 weeks of age, specific pathogen-free (SPF) grade) were purchased from the Beijing Vital River Experimental Animal Co., Ltd. (Vital River, Beijing, China). These animals were kept in an environment at 24 ± 2°C with *ad libitum* access to food and water. All the procedures in this study were performed with the permission of the Animal Ethics Committee and Institute of Materia Medica, Chinese Academy of Medical Science and Peking Union Medical College.

The female SD rats were randomly divided into two groups: the normal pregnancy group and the pregnancy with PH group. After 1 week of adaptive feeding, rats in the pregnancy with PH group were injected subcutaneously in the napes with MCT (Sigma-Aldrich, United States) at a dose of 60 mg/kg to induce PH. Control rats received normal saline (0.9% NaCl). Two weeks later, the rats in each group were placed with males for mating (male rats: female rats = 1:3). Vaginal plugs was the criterion to judge the success of mating. The measurement of pulmonary artery pressure and right ventricle echocardiography were performed; meanwhile, their pulmonary artery and heart were collected, and hematoxylin and eosin (H&E) staining was performed at the experimental endpoint to ensure the establishment of pregnancy with PH rat model. The corresponding groups of rat offspring were established according to the groups of female rats. The growth trend was recorded as they grew up.

### Silencing Myadm of PH Rats Before Pregnancy

Sprague–Dawley (SD) rats (6 weeks of age, SPF grade) were purchased from Beijing HFK Bioscience Co., Ltd. (Beijing, China). These animals were kept in an environment at 24 ± 2°C with *ad libitum* access to food and water. All the procedures in this study were performed with the permission of the Animal Ethics Committee and the Institute of Materia Medica, Chinese Academy of Medical Science and Peking Union Medical College.

The female SD rats were randomly divided into three groups: the normal control, the maternal PH group, and the intervention group. After 1 week of adaptive feeding, rats in the maternal PH group and intervention group were injected subcutaneously in the napes with MCT (Sigma-Aldrich, United States) at a dose of 55 mg/kg to induce PH. Control rats received normal saline (0.9% NaCl). At the same time, shRNA-Myadm was used to knock out this gene in MCT-induced PH female rats in the intervention group through tail vein injection. Two weeks later, the rats in each group were placed with males for mating (male rats: female rats = 1:3). Vaginal plugs was the criterion to judge the success of mating. The right ventricle echocardiography was performed; moreover, their pulmonary artery and heart were collected, and hematoxylin and eosin (H&E) staining was performed at the experimental endpoint to evaluate the effect of silencing Myadm of PH rats before pregnancy. And the interference efficiency of shRNA-Myadm was detected by Western blot analysis and RT-qPCR in their heart and lung. The corresponding groups of rat offspring were established according to the groups of female rats. The growth trend was recorded as they grew up.

### Analysis of Gene Expression Profiles of Heart Tissue of Maternal PH Newborn Rats

Newborn rats (without gender distinction) within 24 h of birth in the normal group and maternal PH group were obtained. Hearts of newborn rats (*n* = 3) in each group were collected, the gene expression profiles of which were detected and analyzed. Total RNA in heart tissue was extracted and measured by NanoDrop ND-1000. One to two μg total RNA from each sample was used to construct a sequencing library, which was detected by Illumina NovaSeq 6000. Then, DEGs were screened, and functional enrichment analysis was performed. This part of the work was finished by Shanghai Aksomics Co., Ltd.

### Sample Collection, Tissue Weighing, and Histology

First, as for the rat offspring in the normal group and maternal PH group, blood was collected by sodium citrate anticoagulant tubes from the left common carotid artery of offspring at their 10 weeks of age and 14 weeks of age. After centrifugation at 5,000 *g* for 10 min at 4°C, the plasma was isolated and stored at −80°C. Then, the offspring were immediately sacrificed. Their body weights and heart weights were recorded. In addition, as for rat offspring in the normal group, maternal PH group, and intervention group, their body weights and organ weights were recorded at their 1, 3, and 6 weeks of age, respectively. Several whole hearts were fixed with 4% paraformaldehyde, embedded in paraffin, and sliced into 5 μm sections, and then hematoxylin and eosin (H&E) staining and Masson’s staining were performed, followed by examination with a light microscope (Nikon). The rest of the hearts were stored at −80°C for molecular experiments. In addition, the cerebrum was obtained and divided into the cerebral cortex and other parts, and then stored at -80°C for molecular experiments.

### Enzyme-Linked Immunosorbent Assay

The plasma samples obtained before were used to detect the expression of endothelin (ET)-1 in the circulatory system using the enzyme-linked immunosorbent assay (ELISA). In addition, total protein was extracted from the heart and cerebral cortex when the offspring were 10 or 14 weeks of age, respectively. Protein concentrations were standardized using Bicinchoninic Acid (BCA) Protein Assay Reagent (Thermo Fisher Scientific). Then, the protein levels of brain natriuretic peptide (BNP) in the heart tissue and hypoxia-inducible factor (HIF)-1α in the cerebral cortex were detected with relevant ELISA kits. All these ELISA kits (Jiangsu Meimian Industrial Co., Ltd., China) were used according to the manual guide.

### Western Blot Analysis

Western blot analysis was used to measure the expression of hypoxia- and inflammation-related proteins in the cerebral cortex of the offspring, as well as the Myadm expression in lungs and hearts of maternal rats. Total protein was extracted from the cerebral cortex of offspring at 10 weeks of age and 14 weeks of age, as well as of offspring at 1, 3, and 6 weeks of age using radioimmunoprecipitation assay (RIPA) lysis buffer (Solarbio) containing protease inhibitor and phosphatase inhibitor cocktails, and the protein concentrations were determined by Bicinchoninic Acid (BCA) Protein Assay Reagent (Thermo Fisher Scientific). Proteins were heated for 5 min at 100°C with the loading buffer, separated on 8% SDS-PAGE gels, and electrotransferred for 1–2 h to cross-link to a polyvinylidene fluoride membrane. After incubation overnight with anti-matrix metalloproteinase-2 (MMP2; 1:1000, Abcam), anti-matrix metalloproteinase-9 (MMP9; 1:1000, Abcam), anti–β-actin (1:4000, Abcam), anti–HIF-1α (1:1000, Abcam), anti–cyclooxygenase-2 (COX-2; 1:1,000, Abcam), anti-high mobility group box 1 (HMGB1; 1:1000, Abcam), anti-inducible nitric oxide synthase (iNOS; 1:500, Proteintech), anti–IL-1β (1:500, Santa Cruz), and anti–IL-6 (1:500, Cell Signaling Technology), membranes were incubated with peroxidase-conjugated secondary antibody for 2 h, and the signals were detected using the enhanced chemiluminescence detection method and quantified by densitometry.

### Real-Time Quantitative Reverse-Transcription Polymerase Chain Reaction

The expression levels of the mRNA encoding HIF-1α in the cerebral cortex and TGF-β1, TGF-β2, MMP2, and TIMP2 in the hearts of the offspring at 10 weeks of age and 14 weeks of age were detected *via* real-time quantitative reverse-transcription polymerase chain reaction (RT-qPCR). In addition, the expression levels of the mRNA encoding HIF-1α, iNOS, IL-1, and IL-6 in the cerebral cortex of the offspring in the normal group, the maternal PH group, and the intervention group; TGF-β1, TGF-β2, MMP2, and TIMP2 in their hearts; as well as Myadm in the hearts and lungs of maternal rats were also detected at their 1, 3, and 6 weeks of age, respectively. Total RNA was extracted from the cerebral cortex and heart tissue using TRIzol (Thermo Fisher Scientific). Then, the quality and quantity of RNA were measured by a NanoDrop (Thermo Fisher Scientific). One microgram of total RNA was reverse-transcribed into cDNA with the Prime Script RT Reagent Kit (TaKaRa) according to the manufacturer’s instructions. TB Green Premix Ex Taq ii (TaKaRa) was used to perform quantitative real-time PCR. The primer sequences used in the present study are listed in Table 1. β-Actin was used as a reference gene. The results were presented as the ratio of detected mRNA to β-actin.

**TABLE 1:** Sequence of oligonucleotides used as PCR primers.

Gene	Position	Primer sequences
β-Actin	Forward	5′-TGT​CAC​CAA​CTG​GGA​CGA​TA-3′
	Reverse	5′-GGG​GTG​TGA​AGG​TCT​CAA​A-3′
HIF-1α	Forward	5′-AAT​CTG​AGG​ACA​CGA​GCT​GC-3′
	Reverse	5′-GCT​GCC​GAA​GTC​CAG​TGA​TA-3′
iNOS	Forward	5′-CAG​CAT​CCA​CGC​CAA​GAA​CG-3′
	Reverse	5′-CAA​TCC​ACA​ACT​CGC​TCC​AAG-3′
IL-1	Forward	5′-AAC​TCG​AGT​GAC​AAG​CCC​GTA​G-3′
	Reverse	5′-GTA​CCA​CCA​GTT​GGT​TGT​CTT​TGA-3′
IL-6	Forward	5′-CCA​CTT​CAC​AAG​TCG​GAG​GCT​TA-3′
	Reverse	5′-GTG​CAT​CAT​CGC​TGT​TCA​TAC​AAT​C-3′
TGF-β1	Forward	5′-GAC​TCT​CCA​CCT​GCA​AGA​CC-3′
	Reverse	5′-GGA​CTG​GCG​AGC​CTT​AGT​TT-3′
TGF-β2	Forward	5′-GCT​CCA​TAC​AGT​CCC​AGG​TG-3′
	Reverse	5′-GTT​CTG​CAA​GCG​AAA​GAC​CC -3′
MMP2	Forward	5′-AGC​TGG​CCC​TGT​TCT​GAC​G-3′
	Reverse	5′-CAC​CCT​CTT​AAA​TCT​GAA​ATC​ACC-3′
TIMP2	Forward	5′-GAA​TAT​CTA​ATT​GCA​GGG​AAG​GC-3′
	Reverse	5′-TGG​GTG​ATG​CTA​AGC​GTG​TC-3′
Myadm	Forward	5′-TAG​TCT​GCA​GCT​GTC​GTC​CT-3′
	Reverse	5′-TGA​TGG​TTG​TAC​GGG​TGA​CTG-3′

### Statistical Analysis

The data extracted from the selected cases were preprocessed to eliminate missing values and outliers. Then, a normal distribution test, Kendall’s tau_b correlation coefficient, and one-way ANOVA were performed to explore the relevance between maternal PH and fetal birth weight using SPSS 21.0.

For the analysis of gene expression profiles from the GEO database, the criteria of a corrected p-value < 0.05 and |log fold change (FC) | > 1 were set to identify DEGs. As for the analysis of gene expression profiles of heart tissues of maternal PH newborn rats, the criteria were set as a corrected p-value < 0.05 and |log fold change (FC) | > 1.5. In addition, p-value < 0.05 was considered statistically significant for functional and pathway enrichment analyses. An interaction score >0.4 was considered statistically significant in the PPI network analysis.

For the present preclinical study, data are presented as the mean value ± SEM or fold change, and these two groups were analyzed by unpaired Student’s t test. Differences with a two-sided p-value < 0.05 were considered statistically significant.

## Results

### PH Patients Show Abnormal Gene Expression Changes Related to the Respiratory System

**FIGURE 1:**
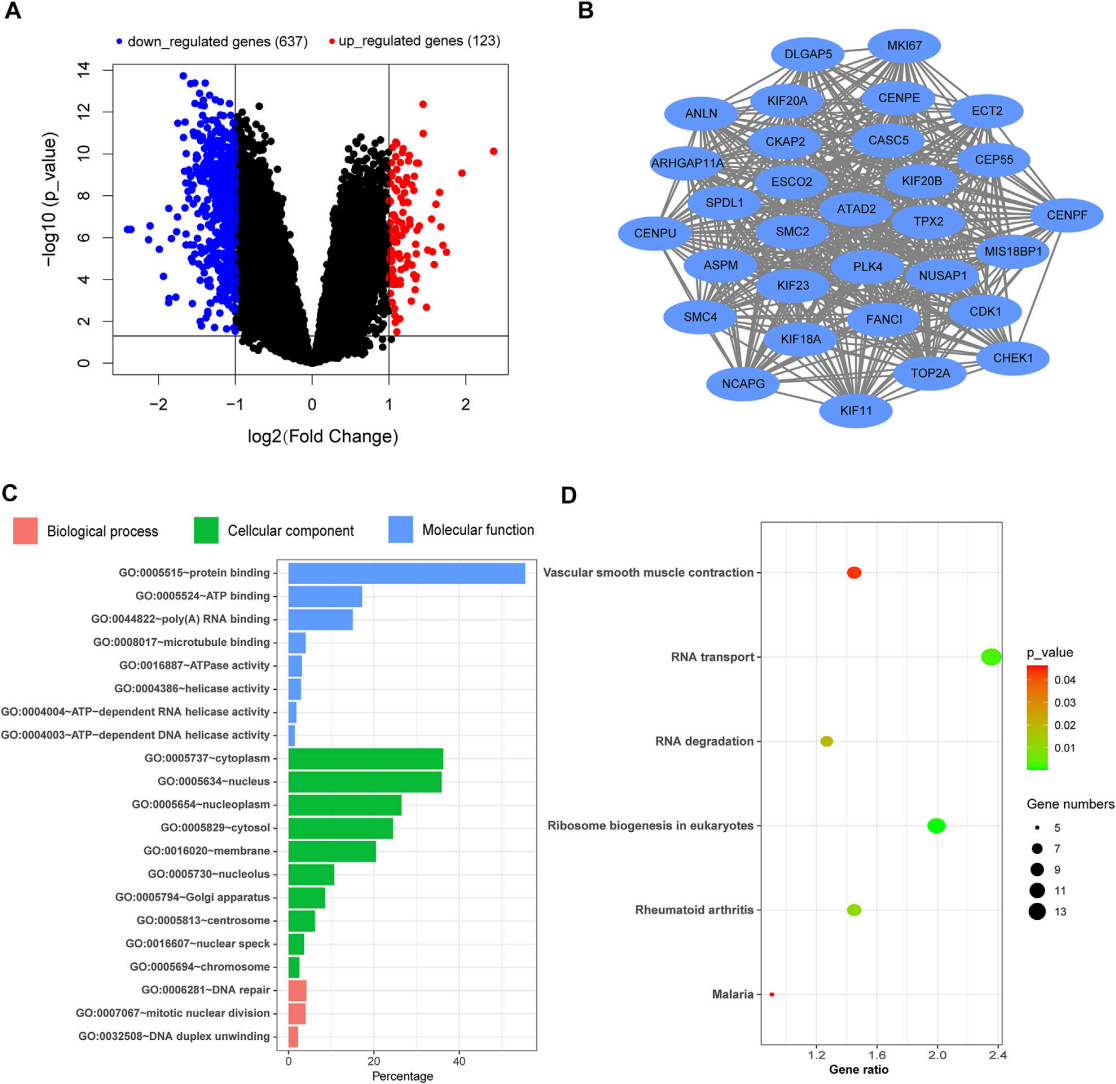
Analysis of the gene expression profile dataset related to pulmonary hypertension (GSE113439). **(A)** Volcano map. The red points represent upregulated genes selected based on the corrected p-value < 0.05 and logFC >1. The blue points represent downregulated genes selected based on the corrected p-value < 0.05 and logFC < −1. The black points represent genes with no significant difference. **(B)** The most significant module was obtained from the PPI network. **(C)** GO enrichment analysis of DEGs. **(D)** KEGG pathway analysis of DEGs. FC, fold change; GO, Gene Ontology; DEGs, differentially expressed genes; KEGG, Kyoto Encyclopedia of Genes and Genomes; PPI, protein–protein interaction.

After analyzing the gene expression profile of PH patients, a total of 760 differentially expressed genes (DEGs) were selected from GSE113439 according to the criteria (corrected p-value < 0.05 and | logFC | > 1), including 123 and 637 genes with upregulated and downregulated expression, respectively. The volcano map is shown in Figure 1A. The most significant module was calculated from the PPI network of DEGs (Figure 1B). The significant GO enrichment results are shown in Figure 1C. The selected DEGs were mainly enriched in the nuclear, membrane, eukaryotic ribosome biosynthesis, and RNA- and DNA-related biological processes. Besides, six key pathways were obtained: “Ribosome biogenesis in eukaryotes,” “RNA transport,” “Rheumatoid arthritis,” “RNA degradation,” “Vascular smooth muscle contraction,” and “Malaria” (Figure 1D). These all indicated that the basic function of the lung cells in PH patients had changed, which affected the function of their respiratory system. These patients were prone to hypoxemia, which further influenced blood and oxygen supply to the placenta while combined with pregnancy.

### Severe PH Patients Are More Likely to Obtain LBW Infants

**FIGURE 2:**
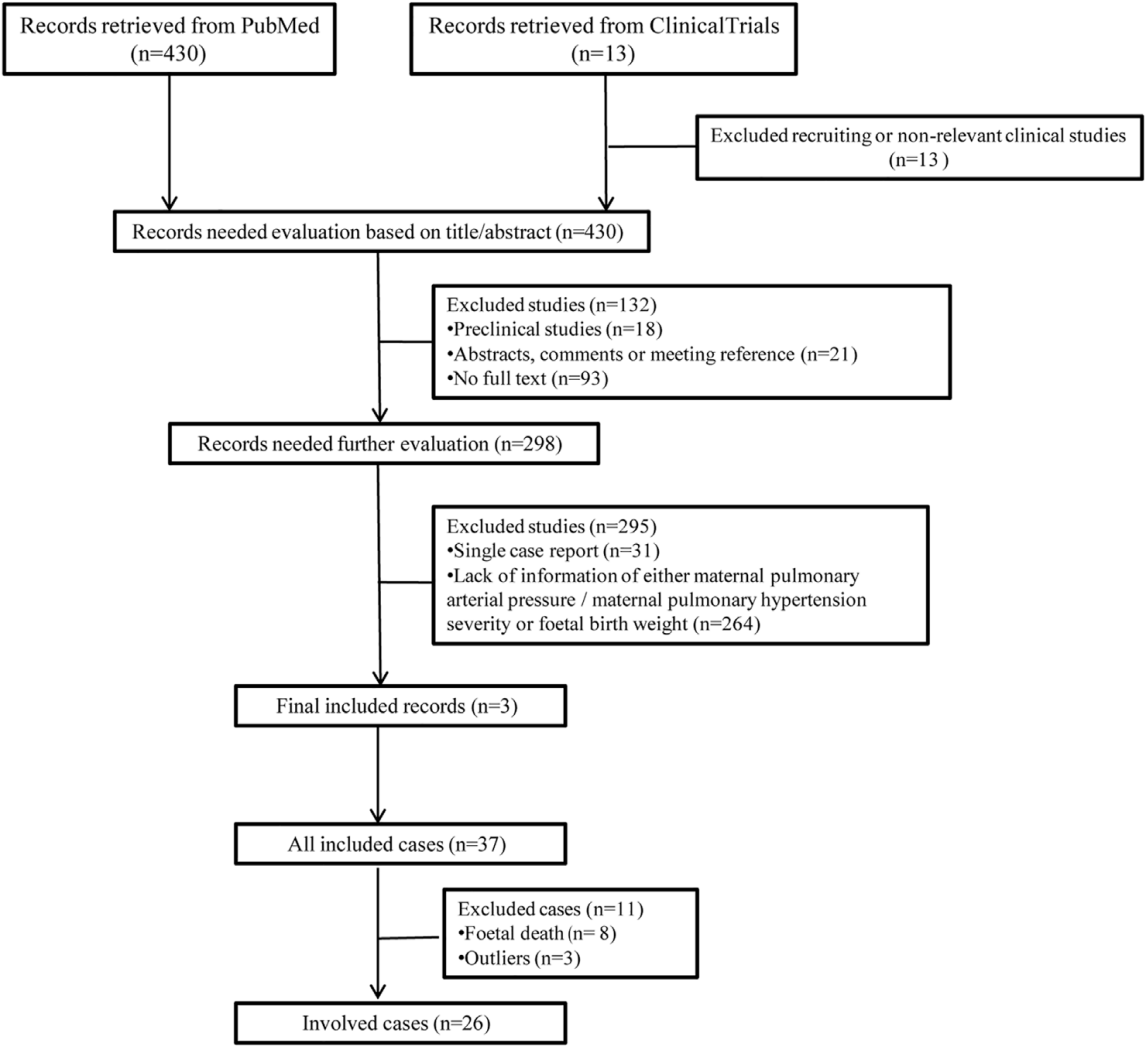
Flowchart of literature selection and case collection.

The detailed process of cases selection is shown in Figure 2. In total, 26 cases were enrolled in the present study, the data of which obeyed a normal distribution. The severity of maternal PH and fetal birth weight was extracted and listed in Table 2. The analysis of Kendall’s tau_b correlation coefficient indicated a significant negative correlation between the severity of maternal PH and fetal birth weight (Table 3). According to the results of a one-way ANOVA (Table 4), maternal severe PH infants presented significantly lower birth weights than both maternal mild and moderate PH infants. Infants of mothers with severe PH were more likely to have a low birth weight.

**TABLE 2:** Included human maternal PH severity and fetal birth weight.

Resources	Case number	Maternal age	Maternal PH severity	Fetal birth weight/g
Monagle et al. (2015)	1	32	Moderate	3,265
	2	25	Moderate	3,040
	3	37	Severe	1,950
	4	32	Mild	2,792
	5	30	Moderate	2,875
	6	33	Moderate	3,715
	7	37	Mild	2,484
	8	30	Severe	1,748
	9	31	Moderate	2,885
	10	26	Moderate	2,713
	11	30	Moderate	2,610
	12	27	Moderate	2,900
	13	36	Moderate	3,130
	14	37	Moderate	3,030
	15	29	Moderate	3,810
Duan et al. (2016)	16	28	Severe	1,740
	17	21	Severe	1,825
	18	25	Severe	2,045
	19	17	Severe	1,690
	20	35	Severe	1,630
	21	26	Severe	1,720
Wang et al. (2018)	22	21	Moderate	2,180
	23	19	Moderate	2,260
	24	33	Mild	2,200
	25	24	Mild	3,600
	26	32	Moderate	3,250

**TABLE 3:** Correlation between human maternal PH severity and fetal birth weight.

Item	Kendall’s tau_b	Maternal PH severity	Fetal birth weight
Maternal PH severity	Correlation coefficient	1	−0.494^a^
	Sig.(2-tail)	—	0.002
Fetal birth weight	Correlation coefficient	−0.494^a^	1
	Sig.(2-tail)	0.002	—

a Correlation is significant at the 0.01 level (2-tailed).

**TABLE 4:** Mean fetal birth weight in each human maternal PH group.

The severity of maternal PH	Fetal number	Mean ± SEM/g
Mild	4	2,769.00 ± 302.22
Moderate	14	2,975.93 ± 124.14
Severe*	8	1,793.50 ± 49.37

*p < 0.05 vs. infants in maternal mild or moderate PH group.

### Human Infants With LBW Show Abnormal Gene Expression Changes Compared With NBW Ones

**FIGURE 3:**
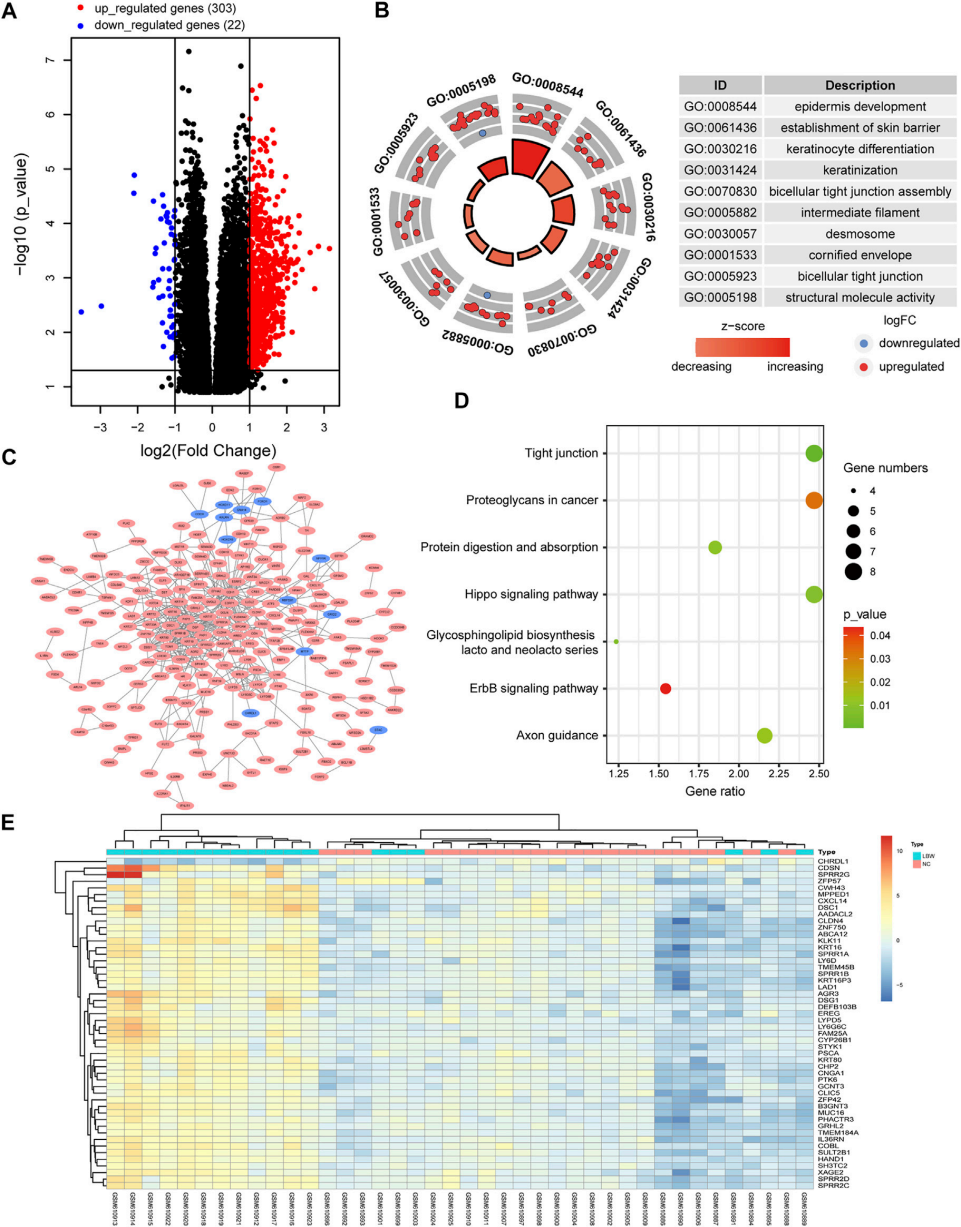
Analysis of the gene expression profile dataset related to human infants with LBW (GSE24818). **(A)** Volcano map. The red points represent upregulated genes selected based on the corrected p-value < 0.05 and logFC >1. The blue points represent downregulated genes selected based on the corrected p-value < 0.05 and logFC < −1. The black points represent genes with no significant difference. **(B)** GO enrichment analysis of DEGs. **(C)** PPI network analysis of DEGs. **(D)** KEGG pathway analysis of DEGs. **(E)** Heat map. The heat map displays the first 50 DEGs with the highest | logFC | using the pheatmap package of R software. Red indicates that genes are expressed at a relatively high level. Blue indicates that genes are expressed at a relatively low level. Yellow is set in the middle. LBW, low birth weight; FC, fold change; GO, Gene Ontology; DEGs, differentially expressed genes; KEGG, Kyoto Encyclopedia of Genes and Genomes; PPI, protein–protein interaction.

Based upon the analysis results, it was started from the perspective of adverse birth outcomes of pregnancy with PH, and the DEGs in LBW infants were screened. A total of 325 DEGs were selected from GSE24818 according to the criteria (corrected p-value < 0.05 and | logFC | > 1), including 303 and 22 genes with upregulated and downregulated expressions, respectively. The volcano map is shown in Figure 3A. The cluster heat map of the former 50 DEGs with the highest | logFC | is shown in Figure 3E. The significant GO enrichment results are shown in Figure 3B. Seven key pathways were obtained: “Tight junction,” “Glycosphingolipid biosynthesis-lacto and neolacto series,” “Hippo signaling pathway,” “Protein digestion and absorption,” “Axon guidance,” “Proteoglycans in cancer,” and “ErbB signaling pathway” (Figure 3D). The PPI network of DEGs was constructed (Figure 3C). According to DEG functional enrichment analysis, the Hippo signaling pathway and ErbB signaling pathway were relative to the development of the cardiac system, while Axon guidance was associated with the nervous system, which indicated the observation direction for the next step.

### Maternal PH Rat Offspring Have Lower Birth Weights, Lower Body Weights, and Slower Growth Rates

**FIGURE 4:**
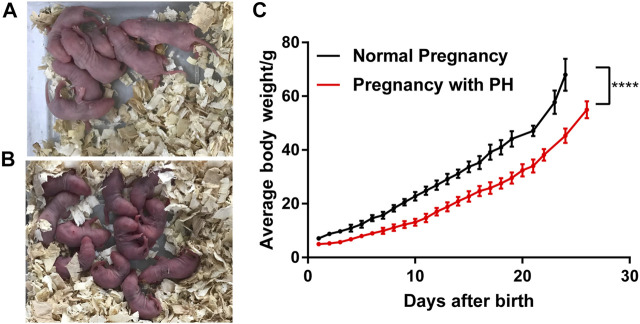
Growth of rat offspring in normal pregnancy and the pregnancy with PH group. **(A)** Offspring in the normal pregnancy group on the third day after birth. **(B)** Offspring in the PH pregnancy group on the third day after birth. **(C)** The growth of average body weight (mean ± standard deviation) in each group was analyzed by paired Student’s t test. ****p < 0.0001 *vs* the normal pregnancy group (*n* = 6 rats per group). PH, pulmonary hypertension.

After the maternal PH rat model was successfully established (Supplementary Figure S1 and Table 4) and the offspring in each group were obtained, the growth status of rat offspring in each group was observed and recorded. The results showed that offspring in the PH pregnancy group (Figure 4B) suffered cyanosis within the initial few days after birth compared with the normal group (Figure 4A), which indicated the emergency of hypoxia. In addition, the offspring in the PH pregnancy group had lower birth weights, lower body weights, and slower growth rates than those in the normal group (Figure 4C).

### Maternal PH Newborn Rats Show Abnormal Gene Expression Changes in Their Hearts

**FIGURE 5:**
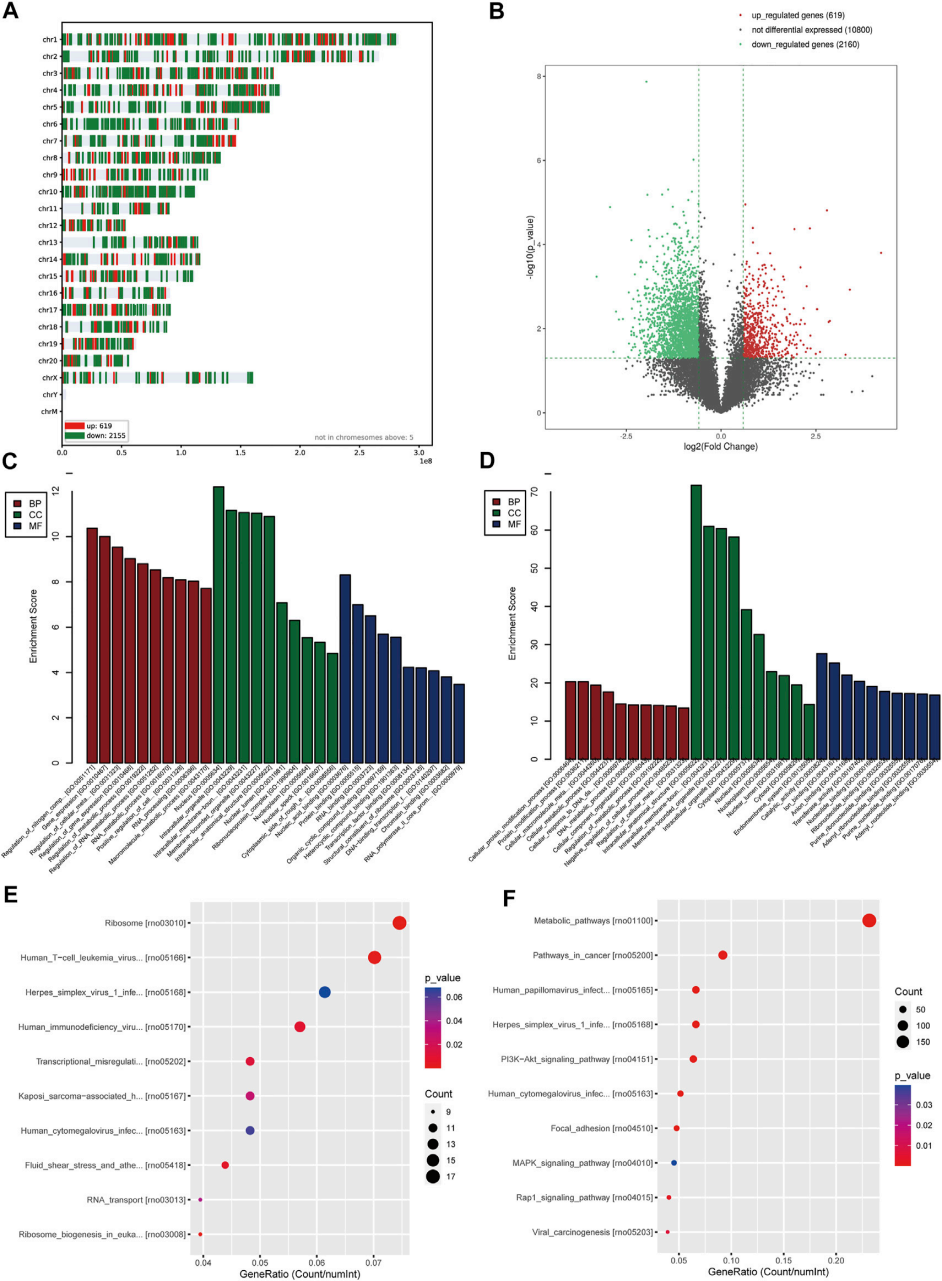
Analysis of the gene expression of heart tissue of maternal PH newborn rats. **(A)** The distribution of DEGs on chromosomes. **(B)** Volcano map. The red points represent upregulated genes selected based on the corrected p-value < 0.05 and logFC >1.5. The green points represent downregulated genes selected based on the corrected p-value < 0.05 and logFC < −1.5. The black points represent genes with no significant difference. **(C)** GO enrichment analysis of upregulated genes. **(D)** GO enrichment analysis of downregulated genes. **(E)** KEGG pathway analysis of upregulated genes. **(F)** KEGG pathway analysis of downregulated genes. GO, Gene Ontology; DEGs, differentially expressed genes; KEGG, Kyoto Encyclopedia of Genes and Genomes.

Totally, 2,779 DEGs were obtained according to the criteria (corrected p-value < 0.05 and | logFC | > 1.5), containing 619 upregulated genes and 2,160 downregulated genes. Figure 5A displayed the distribution of DEGs on chromosomes, and the volcano map is shown in Figure 5B. The significant GO enrichment results are shown in Figure 5C (upregulated genes) and Figure 5D (downregulated genes), respectively. The significant KEGG pathway enrichment results are shown in Figure 5E (upregulated genes) and Figure 5F (downregulated genes), respectively. The results showed that DEGs were mainly enriched in biological processes such as cell metabolism, intracellular protein modification, nucleic acid, and protein binding, as well as pathways related to eukaryotic ribosome biosynthesis, circadian rhythm, DNA replication, and metabolism. These all indicated the abnormal gene expression associated with basic cell function of the heart indeed existed in maternal PH newborn rats.

### Maternal PH Adult Rat Offspring Show Abnormal Cardiac Changes

**FIGURE 6:**
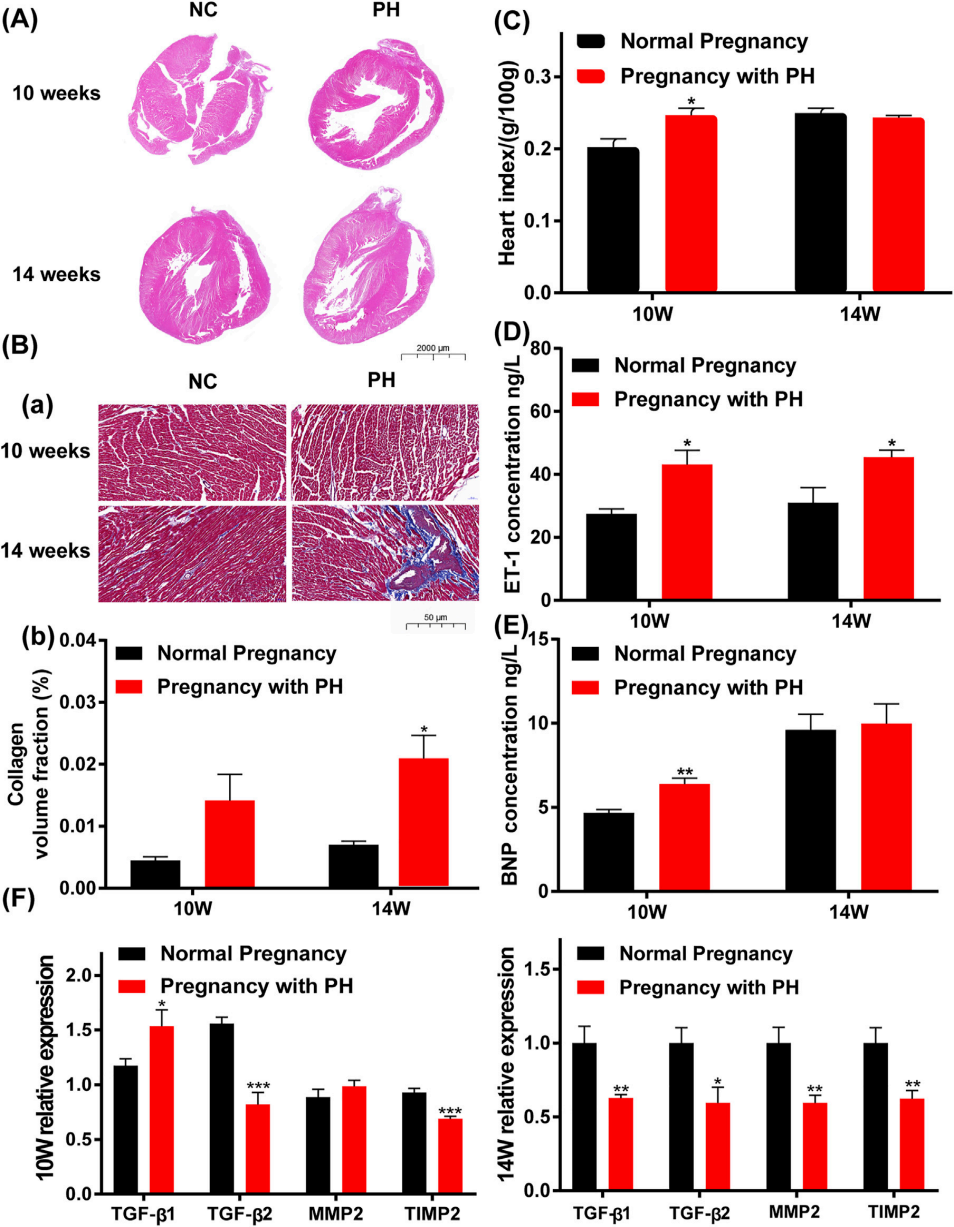
Abnormal cardiac changes in adult rat offspring in the PH pregnancy group. **(A)** Representative H&E-stained hearts. **(B)**. (a) Representative Masson’s staining, Collagen fibers are blue, and muscle fibers are red. (b) Calculation of the collagen volume fraction. The large images show the entire heart (0.5× magnification). The bar indicates 2000 μm. The small images show part of the right ventricular wall (20× magnification). The bar indicates 50 μm. **(C)** The heart index (heart weight/body weight) of offspring at 10 weeks of age and 14 weeks of age in each group. (D) The expression of ET-1 in the plasma in each group at 10 weeks of age and 14 weeks of age was determined by ELISA. **(E)** The expression of BNP in the heart tissue in each group at 10 weeks of age and 14 weeks of age, as determined by ELISA. **(F)** The mRNA expression of TGF-β1, TGF-β2, MMP2, and TIMP2 in the heart tissue of offspring in each group and at each age. *p < 0.05, **p < 0.01, ***p < 0.001 vs. the normal pregnancy group (*n* = 6 rats per group) NC, normal control; PH, pulmonary hypertension.

Offspring rats were sacrificed at 10 and 14 weeks of age. Compared with the normal pregnancy group, the heart index (heart weight/body weight) of offspring in the pregnancy with PH group showed a significant increase in ten-week-old but not in fourteen-week-old rats (Figure 6C). Characteristic molecules were detected to verify the changes in the cardiac system. The level of ET-1 in the plasma of maternal PH offspring increased significantly in both ten-week-old and fourteen-week-old rats (Figure 6D). A significant increase was also found in the expression of BNP in the heart tissues of maternal PH offspring, but only at 10 weeks of age (Figure 6E). In addition, the results of the histological analysis showed that offspring in the PH group manifested thinner right ventricular walls and larger right cardiac chambers at both 10 weeks of age and 14 weeks of age than those in the normal group (Figure 6A), which indicated the emergence of right heart dysfunction. Loss and derangement of myocardial cells were observed in maternal PH offspring, and heavier myocardial fibrosis was found in maternal PH offspring at 14 weeks of age (Figure 6B). However, there was no significant difference in myocardial fibrosis between the two groups at 10 weeks of age (Figure 6B). Then, the expressions of TGF-β1, TGF-β2, MMP2, and TIMP2 were examined. Compared with the normal pregnancy group, offspring in the PH pregnancy group showed significant changes in the mRNA expression of TGF-β1 and TGF-β2, as well as the ratio of MMP2 to TIMP2 at both 10 and 14 weeks of age (Figure 6F) . At the age of 10 weeks, maternal PH offspring showed a significant increase in the mRNA expression of TGF-β1, as well as the significant decreases in mRNA expression of TGF-β2 and TIMP2 compared with normal offspring. However, at the age of 14 weeks, maternal PH offspring showed significant decreases on mRNA expression of TGF-β1, TGF-β2, MMP2, and TIMP2.

### Maternal PH Adult Rat Offspring Show Hypoxia and Inflammation in the Cerebral Cortex

**FIGURE 7:**
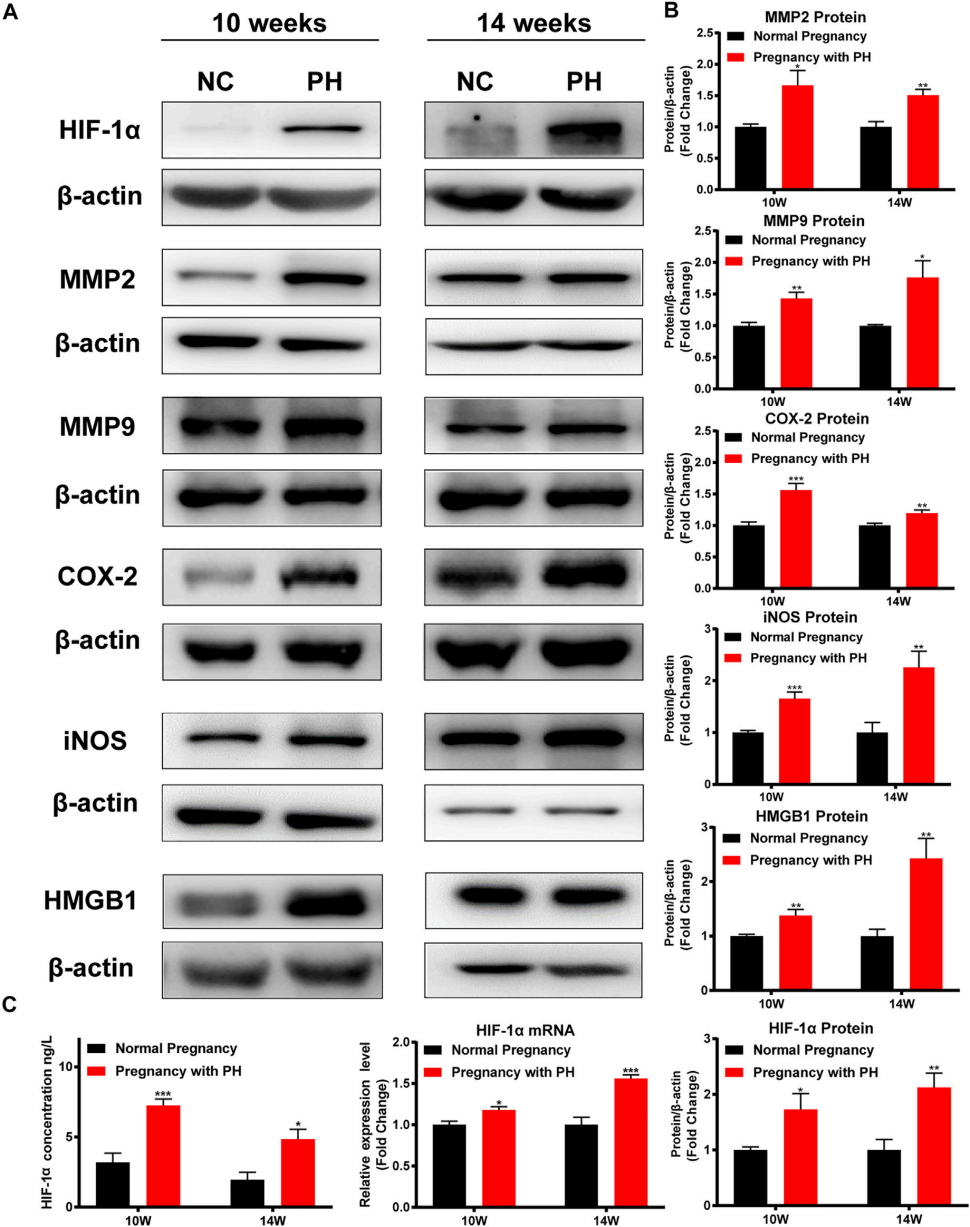
Cerebral hypoxia and inflammation existed in the adult rat offspring of the PH group. **(A)** Western blot analysis of HIF-1α, MMP2, MMP9, COX-2, iNOS, and HMGB1 in the cerebral cortex of offspring in each group at different ages. **(B)** Summary of the data shown in **(A)**. **(C)** The expression of HIF-1α in the cerebral cortex of offspring in each group detected by ELISA, RT–qPCR, and Western blot analysis. *p < 0.05, **p < 0.01, ***p < 0.001 vs the normal pregnancy group (*n* = 6 rats per group). NC, normal control; PH, pulmonary hypertension.

The expression levels of HIF-1α, MMP2, MMP9, COX-2, iNOS, and HMGB1 were examined to identify cerebral changes and inflammation. Maternal PH rat offspring showed higher levels of HIF-1α in the cerebral cortex than the normal group, both at 10 weeks of age and 14 weeks of age (Figure 7C). As shown in Figure 7B, maternal PH rat offspring also showed higher expression of MMP2, MMP9, COX-2, iNOS, and HMGB1 at both 10 weeks and 14 weeks of age than the normal group.

### Silencing Myadm of PH Rats Before Pregnancy Can Alleviate the Low Birth Weight and Low Weight Gain of Maternal PH Rat Offspring

**FIGURE 8:**
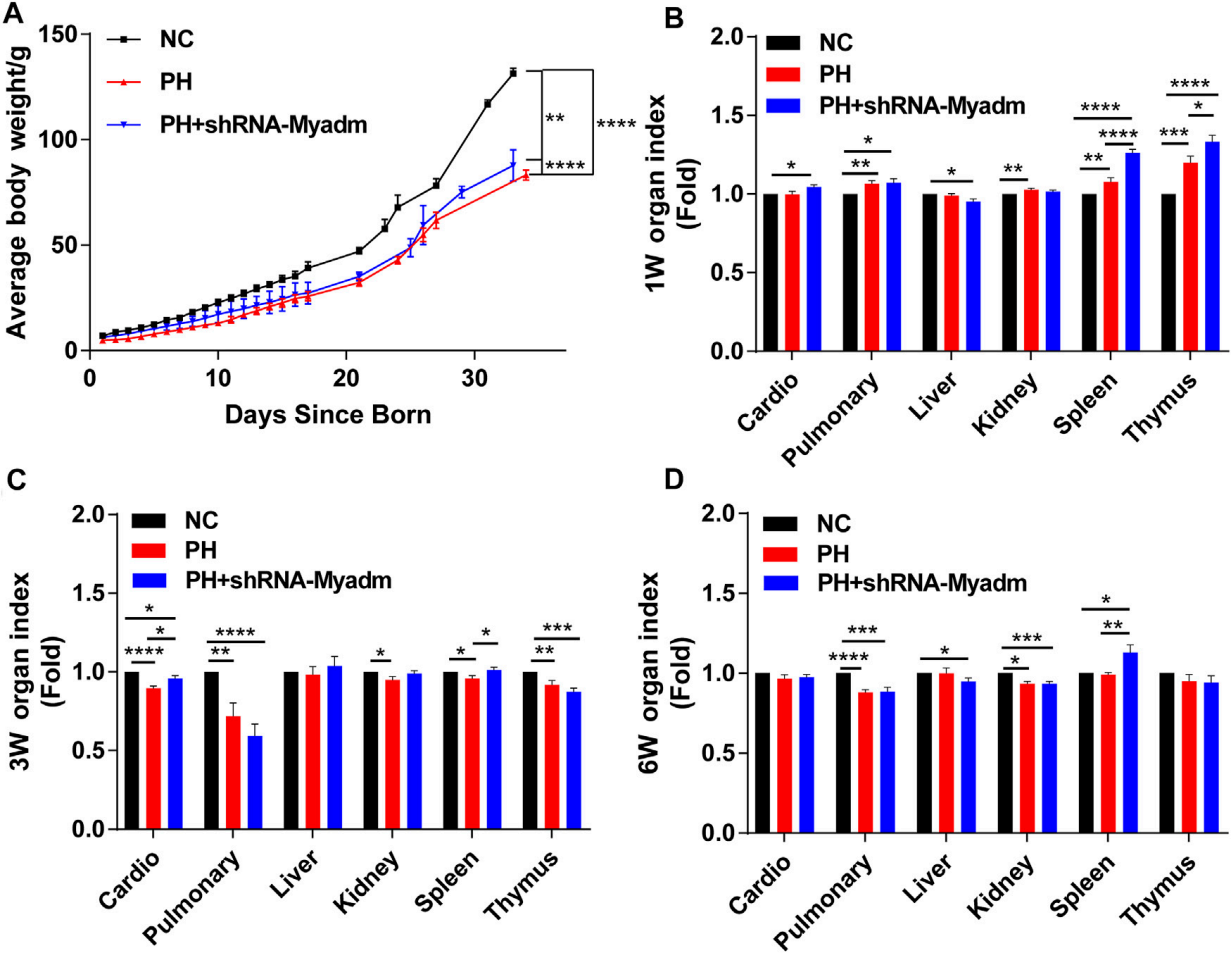
Growth of rat offspring in normal, maternal PH, and intervention groups. **(A)** The growth of body weight (mean ± SD) in each group was analyzed by paired Student’s t test. **(B)** Organ index of offspring in each group at 1 week of age. **(C)** Organ index of offspring in each group at 3 weeks of age. **(D)** Organ index of offspring in each group at 6 weeks of age. *p < 0.05, **p < 0.01, ***p < 0.001, ****p < 0.0001 vs. the normal pregnancy group (*n* = 6 rats per group). NC, normal control; PH, pulmonary hypertension.

The growth status of rat offspring in each group was recorded, and the results showed that silencing Myadm of PH female rats before pregnancy could alleviate the low birth weight and lower body weight of maternal PH rat offspring to a certain degree (Figure 8A). Besides, organ indexes were calculated at their 1 week of age (Figure 8B), 3 weeks of age (Figure 8C), and 6 weeks of age (Figure 8D). Organ indexes of the intervention group underwent significant changes in three ages to a certain extent compared to those of the other two groups.

### Silencing Myadm of PH Rats Before Pregnancy Can Alleviate Abnormal Cardiac Changes of Maternal PH Rat Offspring as They Grow Up

**FIGURE 9:**
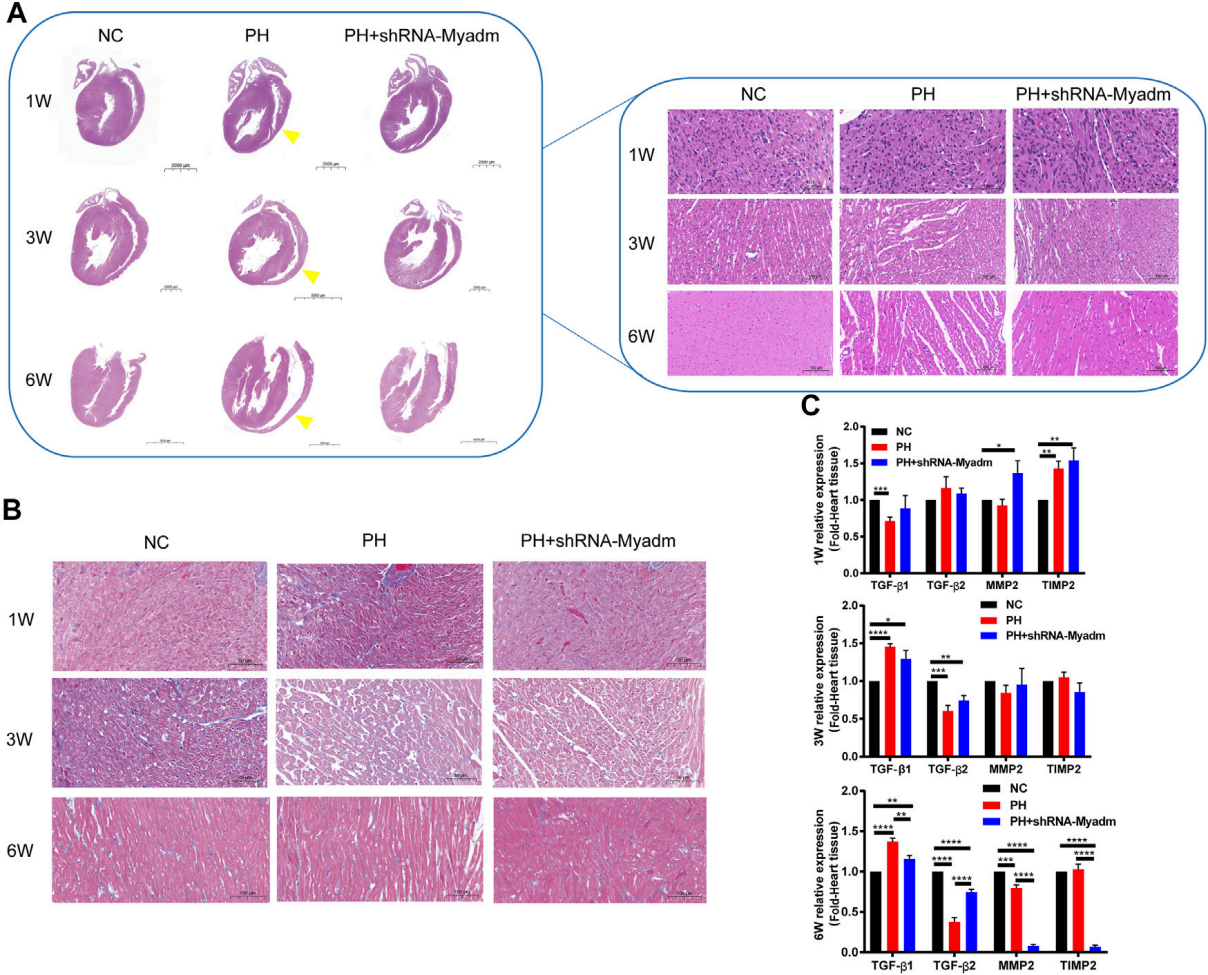
Cardiac changes in rat offspring in normal, maternal PH, and intervention groups. **(A)** Representative H&E-stained hearts of rat offspring at each age in each group. The left images show the entire heart, and the right images show the section of the right ventricular wall. Yellow arrows show thinner right ventricular walls and larger right cardiac chambers of maternal PH rat offspring as they grow up. **(B)** Representative Masson’s staining at each age in each group. **(B)** The mRNA expression of TGF-β1, TGF-β2, MMP2, and TIMP2 in the heart tissue of offspring in each group and at each age. *p < 0.05, **p < 0.01, ***p < 0.001, ****p < 0.0001 vs. the normal pregnancy group (*n* = 6 rats per group). NC, normal control; PH, pulmonary hypertension.

Offspring rats were sacrificed at 1, 3, and 6 weeks of age. Compared with the normal pregnancy group, the results of the histological analysis showed that maternal PH offspring manifested thinner right ventricular walls and larger right cardiac chambers as they grew up than those in the normal group (yellow arrow). However, silencing Myadm of PH rats before pregnancy could alleviate this abnormal cardiac change (Figure 9A). In addition, loss and derangement of myocardial cells were observed in maternal PH offspring as they grew up, which could be alleviated by silencing Myadm of PH rats before pregnancy to some extent (Figure 9B). Then, the expressions of TGF-β1, TGF-β2, MMP2, and TIMP2 in their heart tissue were examined (Figure 9C). Compared with the normal control, maternal PH rat offspring showed a significant decrease in the mRNA expression of TGF-β1 at 1 week of age but significant increases at their 3 and 6 six weeks of age, whose fold change of TGF-β1 expression appeared dynamic increase. But silencing Myadm of PH rats before pregnancy could alleviate this dynamic change. However, the expression change of TGF-β2 in maternal PH rat offspring was opposite to that of TGF-β1, whose fold change showed the dynamic decrease as they grew up, while silencing Myadm could also delay this trend. Meanwhile, the expression changes of MMP2 and TIMP2 showed the same trend as TGF-β2, but silencing Myadm did not seem to work on delaying it as they grew up, especially for their extreme low expression changes at 6 weeks of age.

### Silencing Myadm of PH Rats Before Pregnancy May Alleviate Cerebral Hypoxia and Inflammation of Maternal PH Rat Offspring as They Grow Up

**FIGURE 10:**
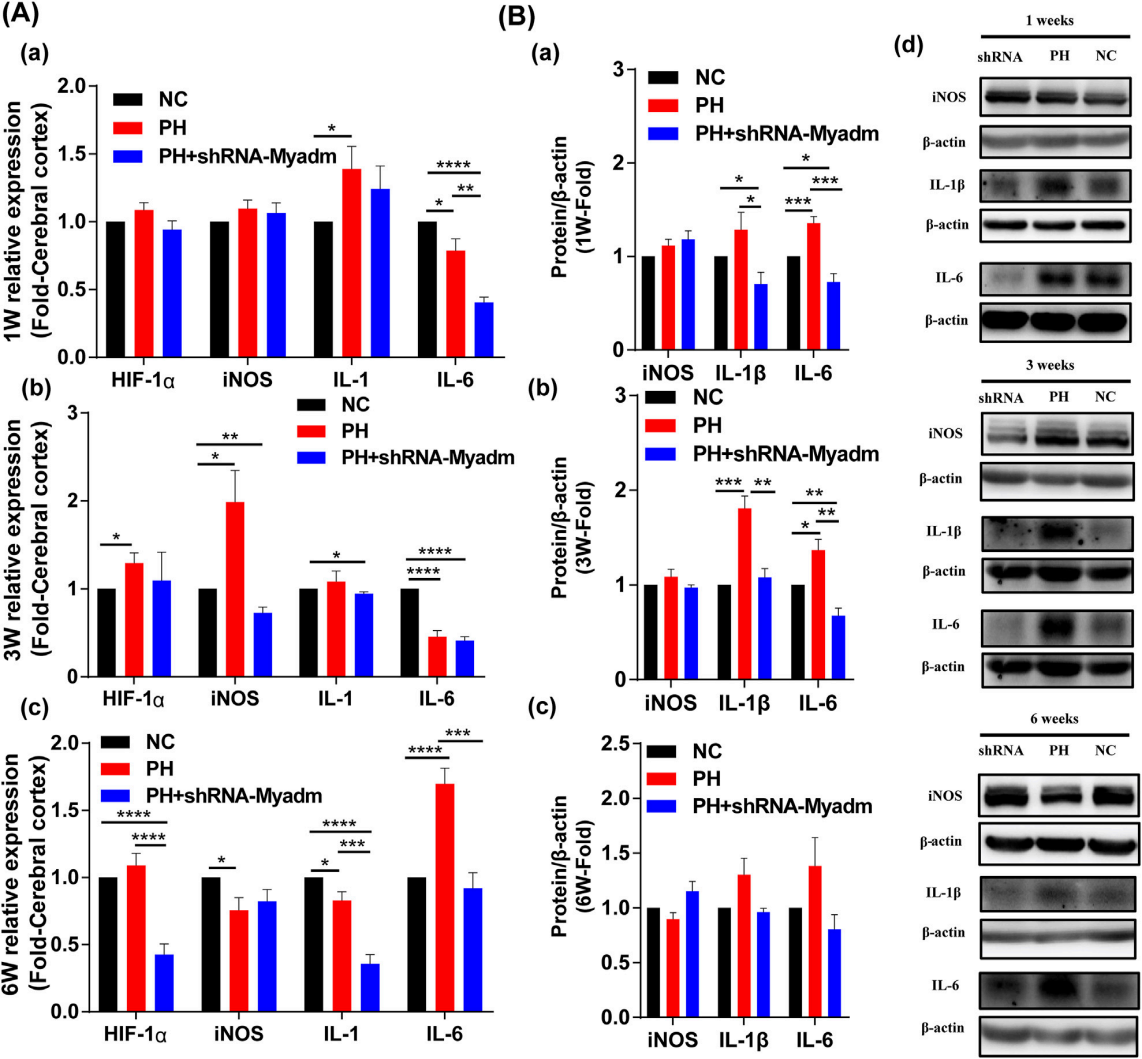
Cerebral hypoxia and inflammation in normal, maternal PH, and intervention groups. **(A)** The mRNA expression of HIF-1α, iNOS, IL-1, and IL-6 in cerebral of offspring in each group at 1 week of age (a), 3 weeks of age (b), and 6 weeks of age (c). **(B)** Western blot analysis of iNOS, IL-1β, and IL-6 in the cerebral of offspring in each group at 1 week of age (a), 3 weeks of age (b), and 6 weeks of age (c). (d) Western blot strips of each group at different ages.*p < 0.05, **p < 0.01, ***p < 0.001, ****p < 0.0001 vs the normal pregnancy group (*n* = 5–6 rats per group). NC, normal control; PH, pulmonary hypertension.

The mRNA expression levels of HIF-1α, iNOS, IL-1, and IL-6 were detected to identify offspring’s cerebral changes and inflammation at 1 week of age (Figure 10A(a)), 3 weeks of age (Figures 10A(b)), and 6 weeks of age (Figures 10A(c)) by RT-qPCR, respectively. Compared with the normal group, the mRNA expression of HIF-1α in the maternal PH group increased but not significantly at their 1 week of age, and increased significantly at their 3 and 6 weeks of age, while silencing Myadm of PH rats before pregnancy could alleviate relative high expression of HIF-1α in the brain of maternal PH offspring. However, the mRNA expression of HIF-1α of rat offspring in the intervention group showed significant decrease compared to the other groups at 6 weeks of age. In addition, the mRNA expression of iNOS in the maternal PH group increased but not significantly at their 1 week of age, and increased significantly at 3 weeks of age, but significantly decreased at 6 weeks of age, while silencing Myadm of PH rats before pregnancy could alleviate the expression change to a certain extent. As for the mRNA expression of IL-1 in brain, maternal PH rat offspring showed the dynamic decrease as they grew up, the same as offspring in the intervention group. As for IL-6, the mRNA expression of IL-6 in the maternal PH group significantly decreased at their 1 and 3 weeks of age, and increased significantly at 6 weeks of age, while silencing Myadm of PH rats before pregnancy could reduce the high expression of IL-6 in the brain. Besides, the protein expression levels of inflammatory factors were also examined, and the results showed that silencing Myadm of PH rats before pregnancy might alleviate brain inflammation of maternal PH rat offspring to some extent (Figure 10B).

## Discussion

In the current study, evidence was provided that maternal PH may induce adverse birth outcomes, implying a further influence on the cardiac and cerebral systems in offspring, and silencing Myadm of PH rats before pregnancy may alleviate these impacts as they grow up. There are three major findings. First, it was demonstrated that PH patients show the gene expression changes of their lungs, which indicated the changes in lung function. These patients are prone to hypoxia, which impacts the intrauterine growth of the fetus. Human maternal PH severity is inversely correlated with fetal birth weight, and women with severe PH are more likely to have surviving infants with LBW, the gene expression profiles of which indicates changes in cardiac and neural development (Mehlen and Furne, 2005; Torres et al., 2010; Lemmens et al., 2011; Hulshoff et al., 2018). In addition, maternal PH rat offspring showing lower birth weight, lower body weights, and slower growth rates indeed exhibit abnormal cardiac changes as well as cerebral hypoxia and inflammation. Therefore, pregnancy with PH may not only lead to a high incidence of adverse pregnancy outcomes but also have a continuing impact on the postnatal growth of surviving offspring, especially for women with more severe PH. Besides, silencing Myadm of PH rats before pregnancy can alleviate the low weight gain and abnormal cardiac–cerebral changes of maternal PH rat offspring to a certain degree.

The first finding shows the inverse correlation between human maternal PH severity and fetal birth weight, indicating that women with severe PH are more likely to acquire newborns with LBW. Based upon the correlation between maternal PH severity and LBW, gene expression profiles of LBW infants were analyzed. Among the remarkably enriched pathways, it was focused on the “ErbB signaling pathway,” “Hippo signaling pathway” and “axon guidance” in the present study. The NGR1/ErbB signaling pathway is the endothelium-controlled system in the heart, the change of which may be an important event leading to ventricular hypertrophy and heart failure (Lemmens et al., 2011). In addition, the change in the TGF-β signaling pathway contained in the “Hippo signaling pathway” was related to myocardial fibrosis (Torres et al., 2010; Hulshoff et al., 2018). These two pathways are both related to the cardiovascular system. In addition, “axon guidance” is associated with the nervous system involving neurons (developing growth cones). For instance, Netrin-1, one of the vital members in this pathway, was identified as playing a crucial role in the development of the nervous system (Mehlen and Furne, 2005). These results all imply that DEGs between LBW and NBW infants were significantly enriched in the related pathways of cardiac and neural development, which might indicate changes in these systems after birth. This provides guidance for this study on continuing effects.

The second finding is that maternal PH rat offspring with apparently lower birth weight, lower body weights, and slower growth rates compared with normal offspring during the period of observation present pathological changes in the cardiac–cerebral system at 10 weeks and 14 weeks of age, which equals five- to seven-year-old children (Andreollo et al., 2012).

Maternal PH rat offspring had lower birth weight, and they showed cyanosis for the initial few days after birth compared with those in the normal pregnancy group, which indicated the emergence of hypoxia, and they maintained continuously lower body weights as they grew up. More concretely, a previous animal experiment reported that IUGR rat offspring caused by maternal protein restriction showed significantly low body weights at first; however, they exhibited catch-up growth from 8 weeks of age to 12 weeks of age, even exceeding the weight of the normal offspring at the same age. Then, the growth rate seemed to slow down, and at 24 weeks of age, the body weight of the offspring was significantly lower than that of the normal offspring (Lim et al., 2006). Whether maternal PH rat offspring show a catch-up growth trend still needs longer-term observation.

For the cardiac system, the impact of maternal PH on rat offspring is shown by an abnormal heart index (heart weight/body weight) as well as the histological and molecular expression changes. Maternal PH rat offspring showed a thinner right ventricular wall and larger right cardiac chamber at both 10 weeks of age and 14 weeks of age than the normal group, which appeared to be one indication of PH–right heart dysfunction. This aligns with the study of Anderson et al. after considering conditions of maternal PH. They reported right ventricular (RV) filling and ejection abnormalities in intrauterine growth restriction (IUGR) young adult baboons using cardiac magnetic resonance imaging (Kuo et al., 2017). Previous studies showed that the adverse intrauterine environment caused by inflammation or nicotine could easily lead to cardiac remodeling or myocardial fibrosis in rat offspring (Chou and Chen, 2014; Chen et al., 2015). It seems that the maternal PH–induced intrauterine environment may also lead to abnormal cardiac changes similar to the loss and derangement of myocardial cells, even myocardial fibrosis. However, a significant increase was only observed in the heart index (heart weight/body weight) and the level of BNP in heart tissue in ten-week-old maternal PH rat offspring compared with normal offspring. These two indices all remained at a higher level at 14 weeks of age compared with those at 10 weeks of age, which may cause a negative result at this age. Additionally, the catch-up growth trend at that period and individual differences may be responsible for this difference. A previous study reported that catch-up growth of IUGR offspring might increase the risk of cardiopulmonary disease as they grew up, the control of which through dietary regulation after birth could protect the cardiopulmonary system, but increase the risk of cognitive impairment (Yan et al., 2019). Hence, it is important to find reasonable methods of postpartum feeding.

The above mentioned findings show that maternal PH–induced impacts continue to affect postnatal growth. It was also suggested that maternal PH has a continuing effect on the growth of offspring, from the intrauterine to the postnatal. However, how does it work? Patients with PH are easily accompanied with hypoxemia, and the previous study indicated that maternal circulatory hypoxia might lead to abnormal placental structure and functions, which might result in developmental changes such as the decrease of contractile force and cardiac output of the fetal heart, and cause complications such as fetal distress and LBW (Bhatla et al., 2003). In addition, gene expression profile analysis of LBW infants may provide possibly changed molecules and pathways. It was speculated that the DEG-enriched pathways play a role in the changes during the postnatal growth period, which are likely to be potential therapeutic directions. The Hippo signaling pathway was highlighted in this study. TGF-β is a widely studied profibrotic factor in the heart. In particular, the TGF-β2–mediated imbalance of MMP2 and TIMP2 also contributes to myocardial fibrosis (Yan et al., 2019). Besides, TGF-β is accepted as a bifunctional regulator that either stimulates or inhibits cell proliferation, which also regulates a variety of key events in normal development and physiology (Morikawa et al., 2016). In the present study, heavier myocardial fibrosis was found in maternal PH offspring at 14 weeks of age than in those at 10 weeks of age. However, the profibrotic factor TGF-β1 appeared in the opposite expressed trend. It was speculated that it may associate with the catch-up growth. The significant increase in TGF-β1 in maternal PH rat offspring at 10 weeks of age may imply catch-up growth due to heart development deficiency, such as a decreased number of myocardial cells, which seems to be a kind of protection of compensatory. However, the previous study pointed out that an adverse intrauterine environment may permanently reduce the number of cells or functional units in vital organs, which in turn would affect postnatal development (Lim et al., 2006). Apart from that, as was mentioned before, even there was a catch-up growth in IUGR offspring, they still got back to the lower body weight at the final observation time compared with the normal group (Lim et al., 2006). Thus, it was suggested that at 14 weeks of age, the remarkable decrease in TGF-β1 probably reflects the real deficiency caused by the adverse intrauterine environment. Furthermore, as for the balance between MMP2 and TIMP2, the decrease in MMP2/TIMP2 also verified the heavier myocardial fibrosis in maternal PH rat offspring at 14 weeks of age.

According to the appearance of cyanosis in maternal PH rat offspring in their initial few days after birth, hypoxia and inflammatory factors in the cerebral cortex were subsequently detected. There is a close relationship between hypoxia and inflammation. HIF-1α regulates the expression of a variety of pathways, such as COX-2, MMP2, and iNOS (Ran et al., 2005; Wang et al., 2012). They all showed significant increases in maternal PH offspring. MMP9, which is involved in many inflammatory processes, is related to the development of many brain diseases, the remarkable increase of which indicated abnormal cerebral changes in maternal PH rat offspring in the present study (Li et al., 2016). In addition, HMGB1 can act as a pro-inflammatory factor, promoting brain damage under pathological conditions (Sun et al., 2019). A significant increase in HMGB1 in the cerebral cortex was a proof of brain changes. These results all imply the impact of maternal PH on the cerebral system of rat offspring to some extent. Moreover, these results suggest that anti-inflammatory or targeting-related pathways may be one of the potential therapeutic strategies.

The third finding shows that silencing Myadm of PH rats before pregnancy can alleviate the low weight gain and abnormal cardiac–cerebral changes of maternal PH rat offspring to a certain degree.

The previous studies found that overexpression of Myadm could induce the remodeling of pulmonary artery and increase pulmonary artery resistance (Sun et al., 2016; Sun et al., 2020). In the present study, shRNA-Myadm was used to knock out this gene in MCT-induced PH female rats, and found that silencing Myadm before pregnancy could alleviate offspring’s low birth weight and low body weight caused by maternal PH to some degree. In addition, the pathological changes of cardiac–cerebral system caused by maternal PH, including enlarged right heart cavity, loss of cardiomyocyte, abnormal heart index, as well as cerebral cortex hypoxia and inflammatory state, were corrected to a certain extent. It is noticed that as for the expression of inflammatory factors in maternal PH rat offspring’s brain, different inflammatory factors showed different expression trends at different ages, which may indicate that different inflammatory factors play a major role at different ages and stages. However, even if silencing Myadm of PH rats before pregnancy can reduce the expression of inflammatory factors to a certain extent, their expressions are also lower than those in normal group. It is speculated that silencing Myadm of PH rats before pregnancy may have a certain effect on the function of the offspring’s immune system.

The limitations in the present study should also be noted here. First, the collected cases of pregnancy with PH from the public database lack the information about other factors that may affect the birth outcome of the offspring, including the mother’s education level and social status. And the number of cases involved in the analysis is not large enough. Apart from that, there are no gene expression profiles of maternal PH infants and normal newborns. The analysis of the gene expression profiles of infants with the same birth outcome as LBW from the perspective of maternal PH infants’ birth outcomes is indirect. Moreover, the existence of hypoxia and inflammatory in rat offspring’s cerebral cortex as well as the abnormal cardiac changes were only detected, while further studies on detailed change sites and concrete mechanisms of these two aspects are definitely needed. In addition, the MCT-induced PH model has the advantages of simple technology, repeatability, good stability, and low cost; however, PH has a complex pathogenesis, and the MCT-induced PH model better simulates clinical pulmonary vascular inflammation–related PH (Nogueira-Ferreira et al., 2015). There are also other kinds of PH, such as PAH and CTEPH, combined with pregnancy. Future observations on different PH models are necessary. However, the PH rat model does not fully represent the human PH condition; the present study can provide some relevant reference to some extent, and further follow-up study on the long-term effect of human maternal PH on offspring’s development after birth is still important. Finally, the long-term effect of maternal PH on the whole offspring was focused for the first time in the present study, and the cardiac and cerebral changes in half male and half female offspring were examined. In the Supplementary Material, the data of male and female offspring were separated to give statistics considering preliminarily sex dependency. The changes in only male or only female offspring are basically consistent with those in the whole offspring; however, it seems that maternal PH affects offspring of different genders with different degrees (Supplementary Figures S3–S6). In the effect of maternal PH on the growth of offspring, the specific differences due to gender differences and the role of sex hormones need to be further studied in the future.

## Conclusion

Maternal PH affects the cardiac–cerebral system of the offspring, showing adverse birth outcomes such as LBW and lower body weight as they grow up in the rat model. Women with severe PH are more likely to give birth to LBW infants and should better pay attention to their children’s growth. Myadm has the potential as a target for prepregnancy treatment and is worth further study. The evidence on the long-term effects of maternal PH in rat offspring was presented for the first time, and the effects of Myadm on pregnancy with PH were explored which provides further reference for the maternal and infant outcomes of pregnancy with PH.

## Data Availability

The datasets presented in this study can be found in online repositories. The names of the repository/repositories and accession number(s) can be found in the article/Supplementary Material.
