# Supplementary figures and images for: Targeting Myadm to Intervene Pulmonary Hypertension on Rats Before Pregnancy Alleviates the Effect on Their Offspring’s Cardiac-Cerebral Systems
Source: Front Pharmacol. 2022 Jan 18;12:791370. doi: 10.3389/fphar.2021.791370 (PMC8804385; doi:10.3389/fphar.2021.791370)

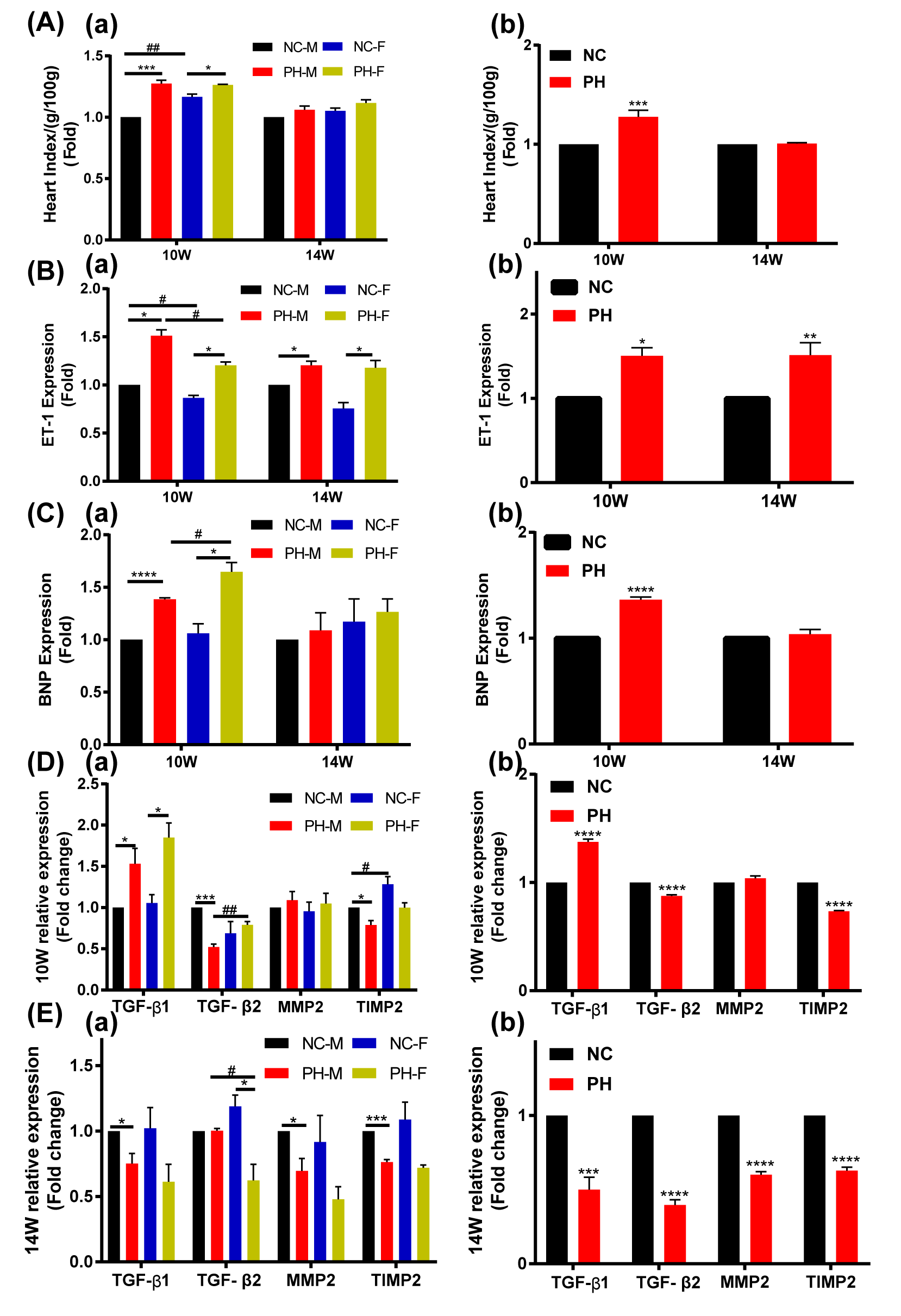

Supplement: Supplementary file 2 [file Image6.TIF]

## Slide 1
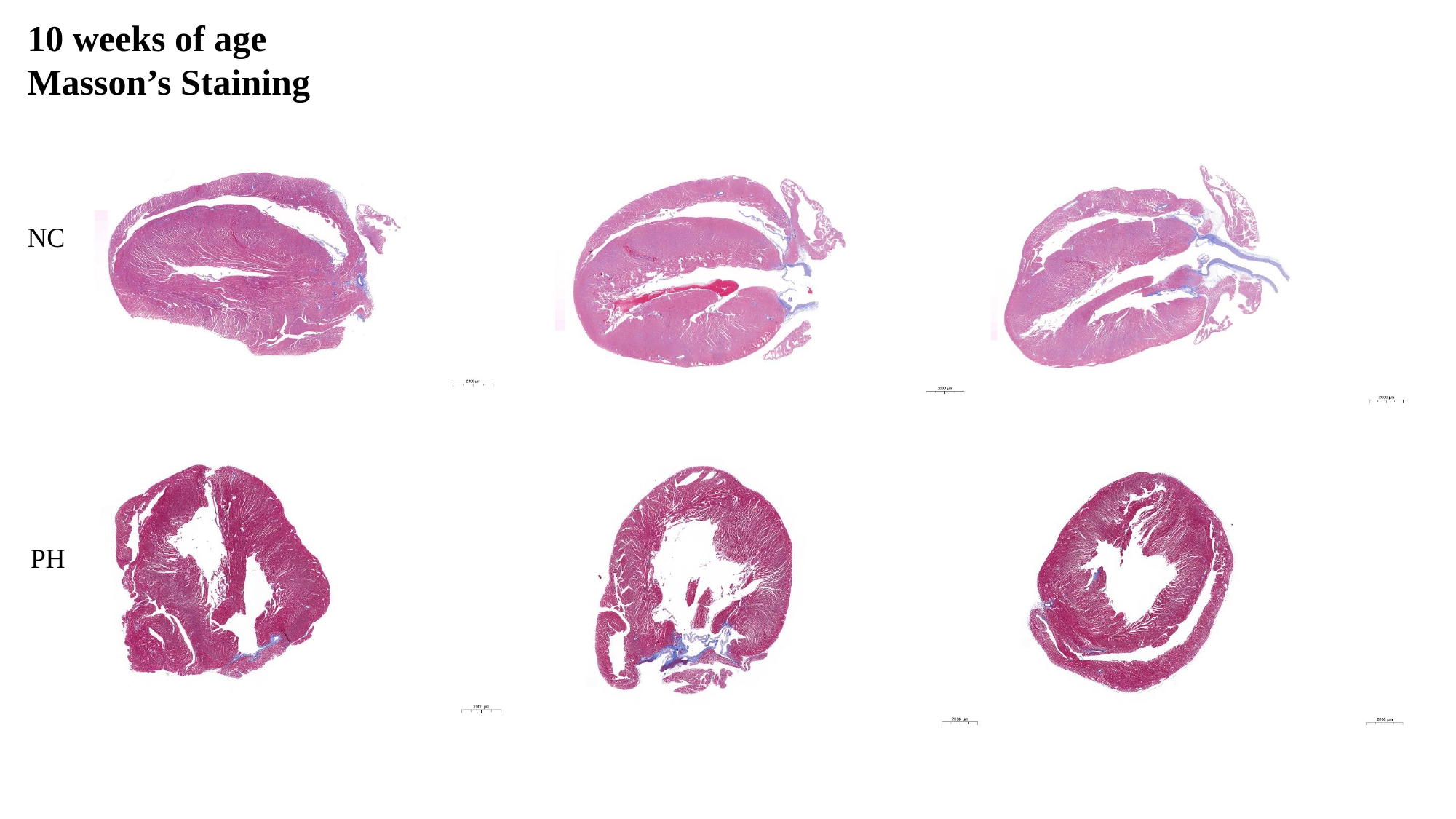

10 weeks of age Masson’s Staining
NC
PH

## Slide 2
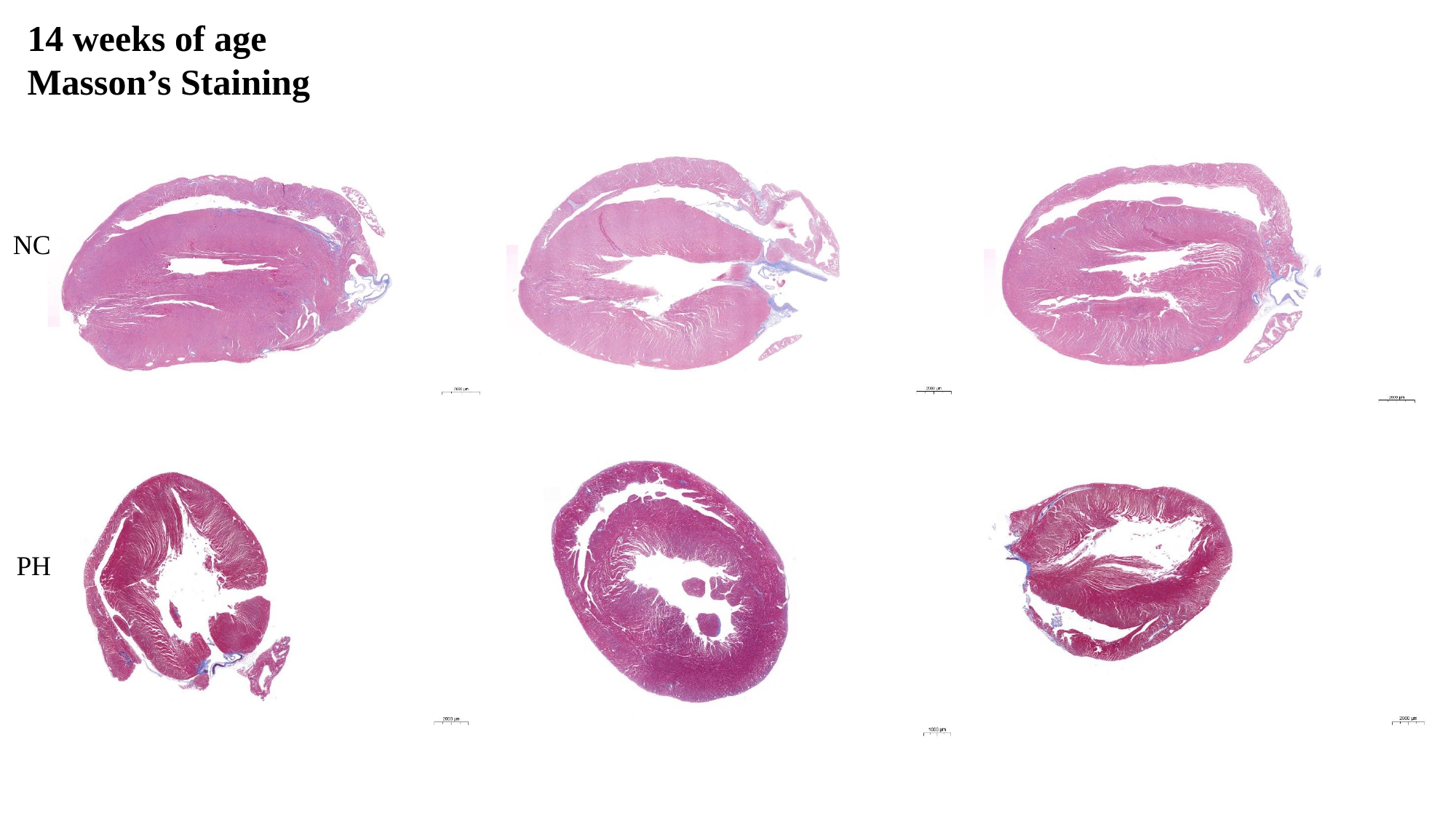

14 weeks of age Masson’s Staining
NC
PH

Supplement: Supplementary file 3 [file Presentation1.PPTX]

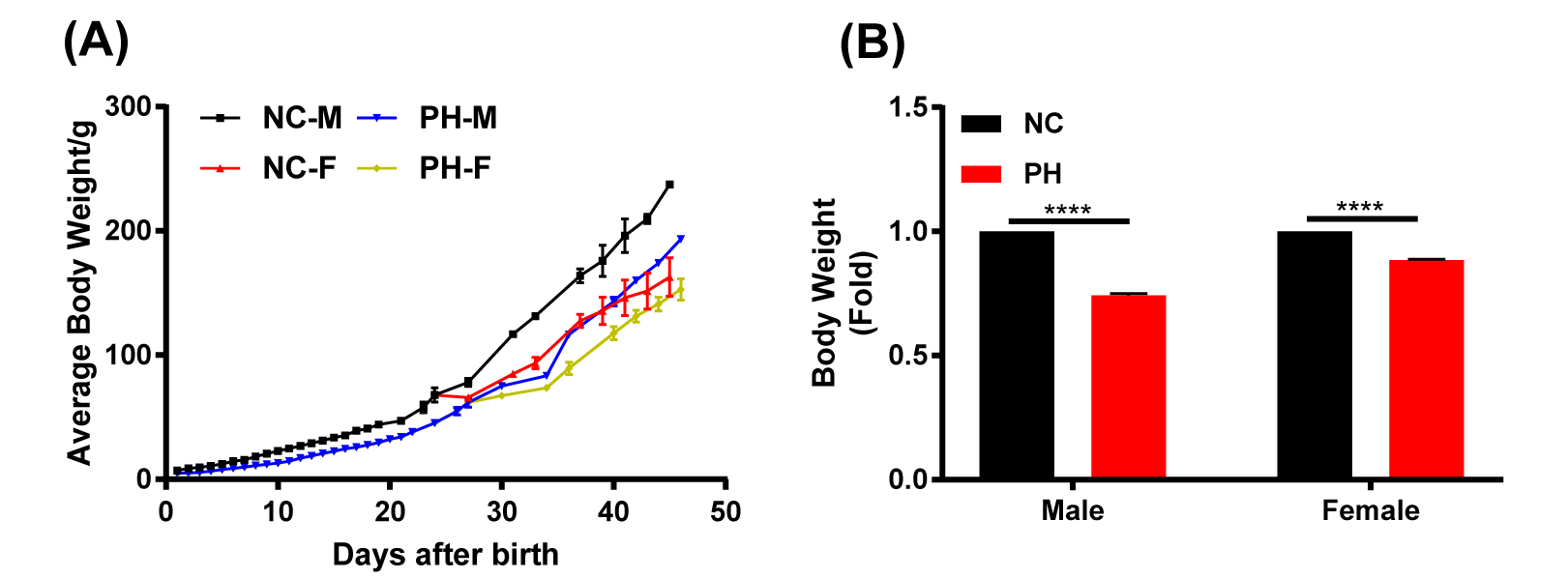

Supplement: Supplementary file 4 [file Image3.TIF]

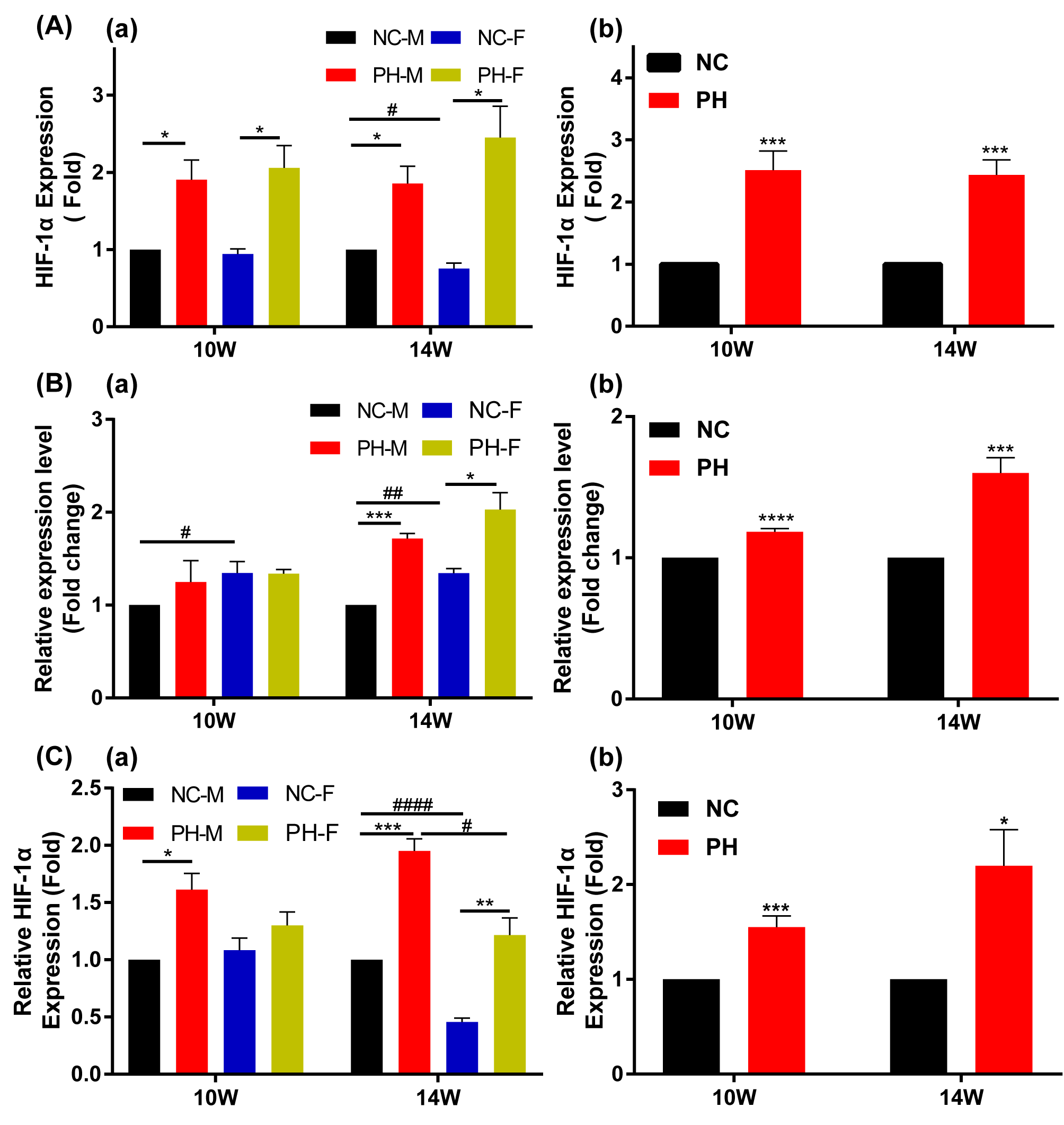

Supplement: Supplementary file 5 [file Image4.TIF]

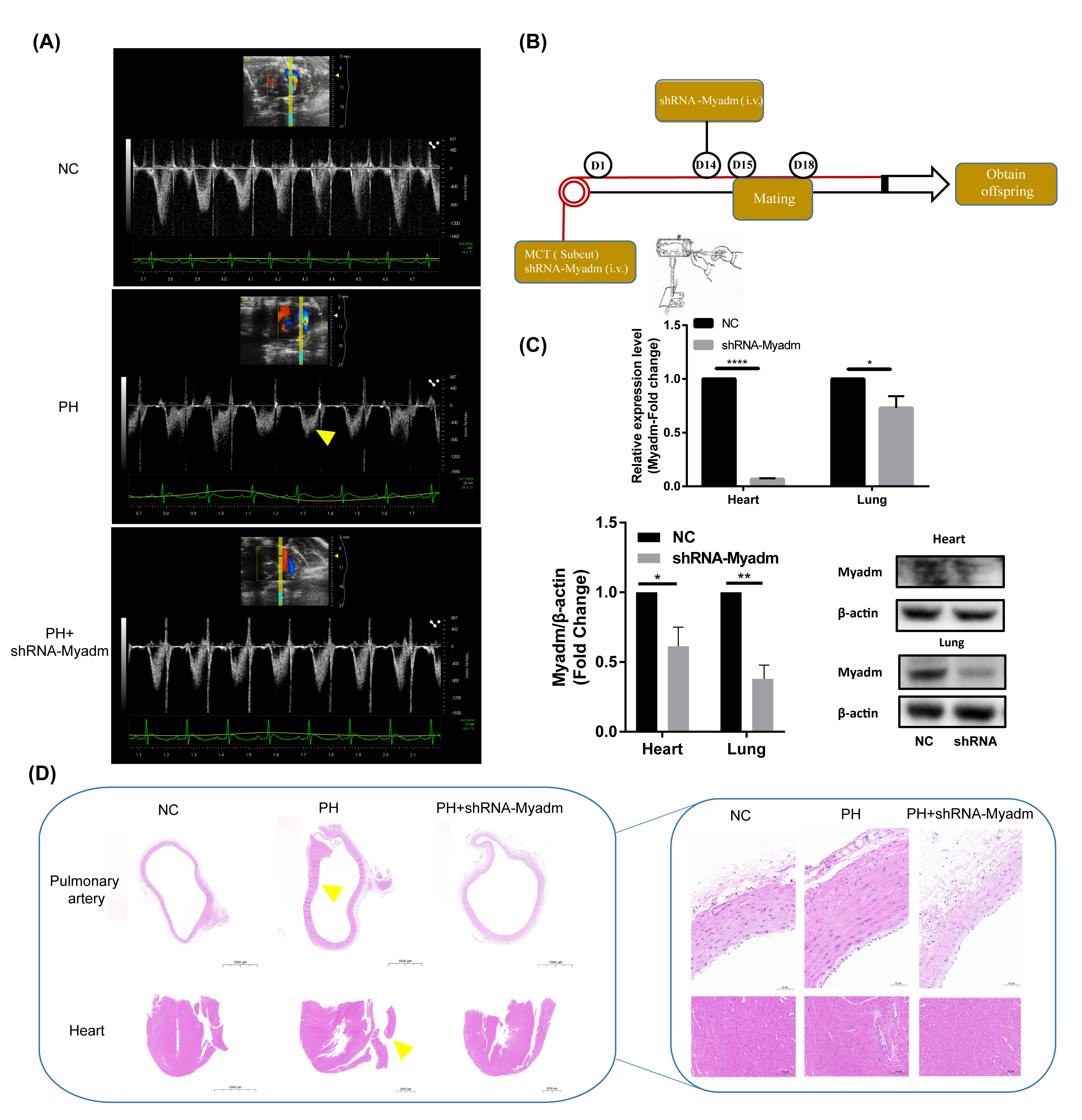

Supplement: Supplementary file 6 [file Image2.TIF]

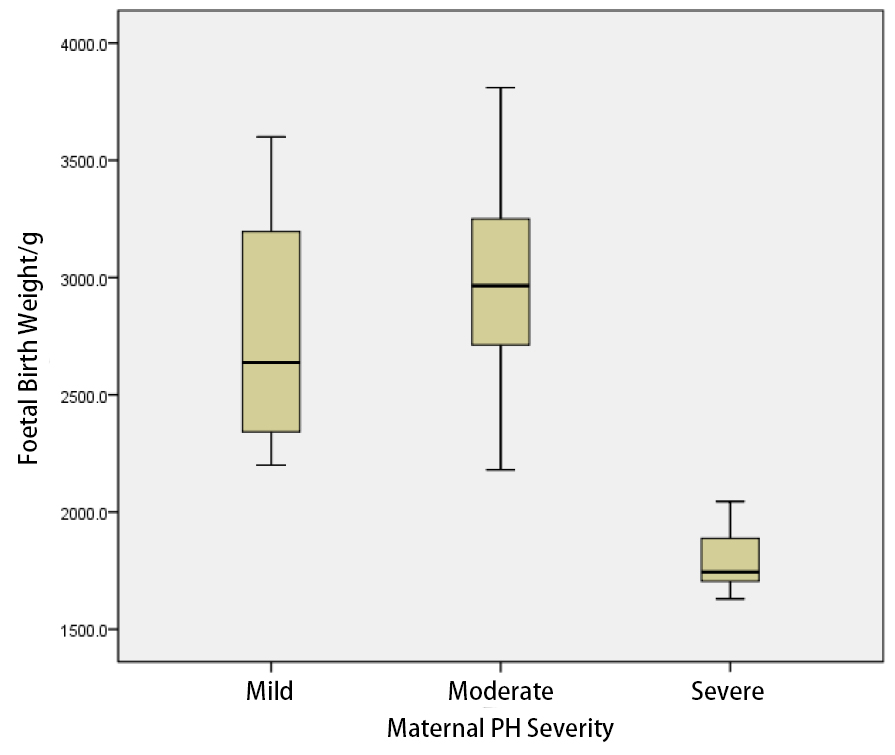

Supplement: Supplementary file 7 [file Image7.JPEG]

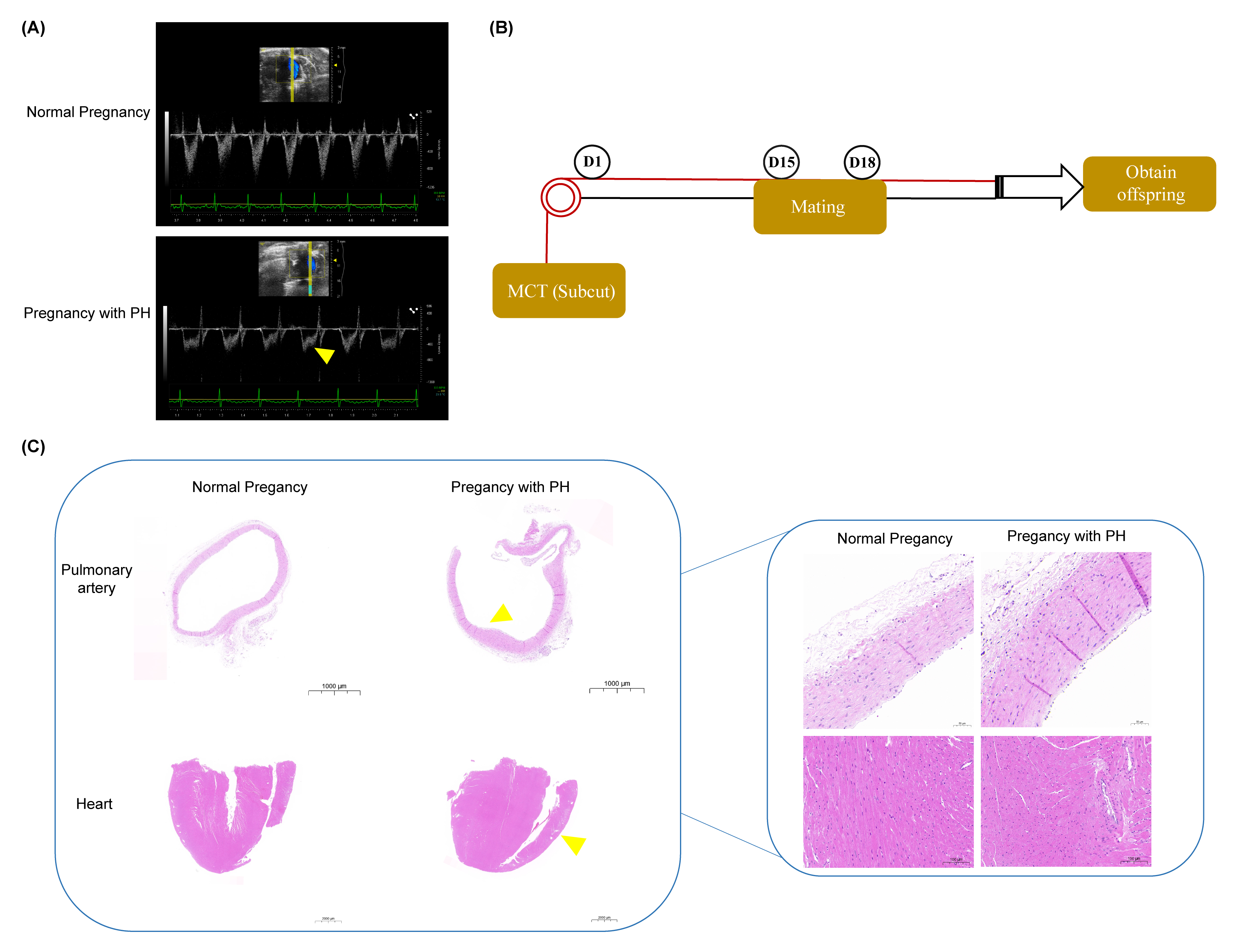

Supplement: Supplementary file 8 [file Image1.TIF]

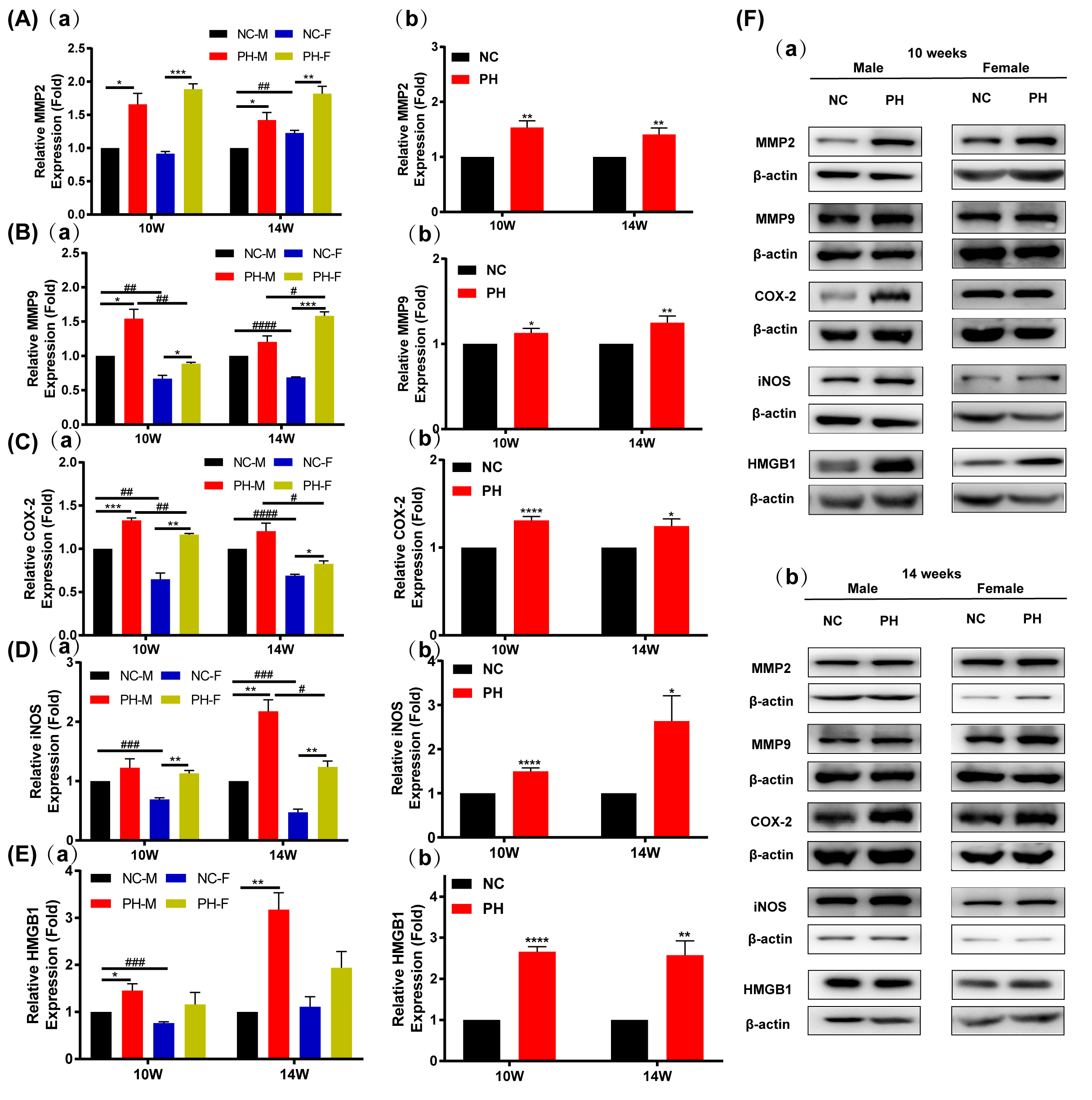

Supplement: Supplementary file 15 [file Image5.TIF]

## Slide 1
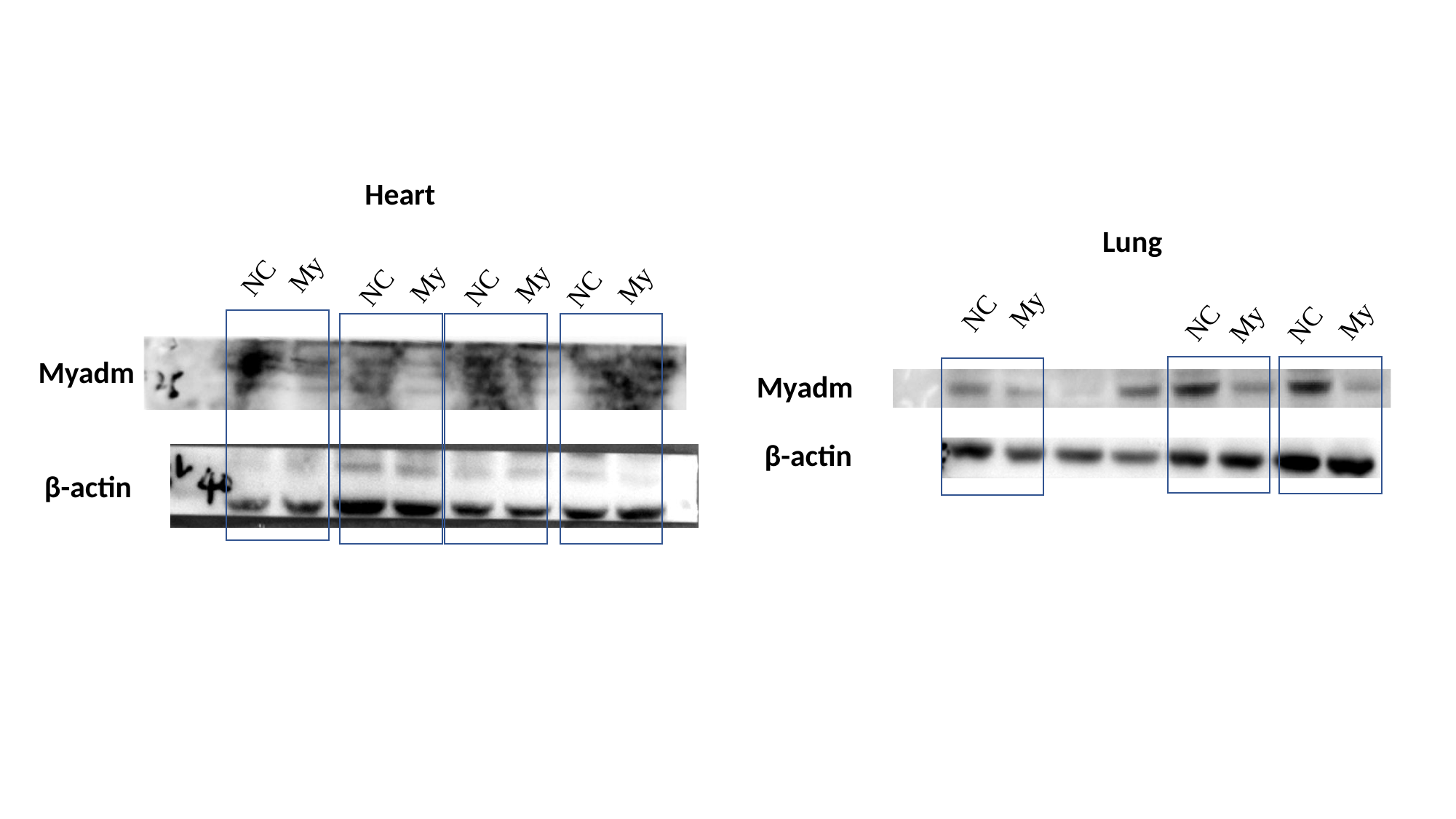

Heart
My
My
My
My
My
Lung
My
My
NC
NC
NC
NC
NC
NC
NC
Myadm
Myadm
β-actin
β-actin

Supplement: Supplementary file 16 [file Presentation7.PPTX]
