# Supplementary material for: Targeting Myadm to Intervene Pulmonary Hypertension on Rats Before Pregnancy Alleviates the Effect on Their Offspring’s Cardiac-Cerebral Systems
Source: Front Pharmacol. 2022 Jan 18;12:791370. doi: 10.3389/fphar.2021.791370 (PMC8804385; doi:10.3389/fphar.2021.791370)
Supplement: Supplementary file 9 [file Presentation4.PPTX]

## Slide 1
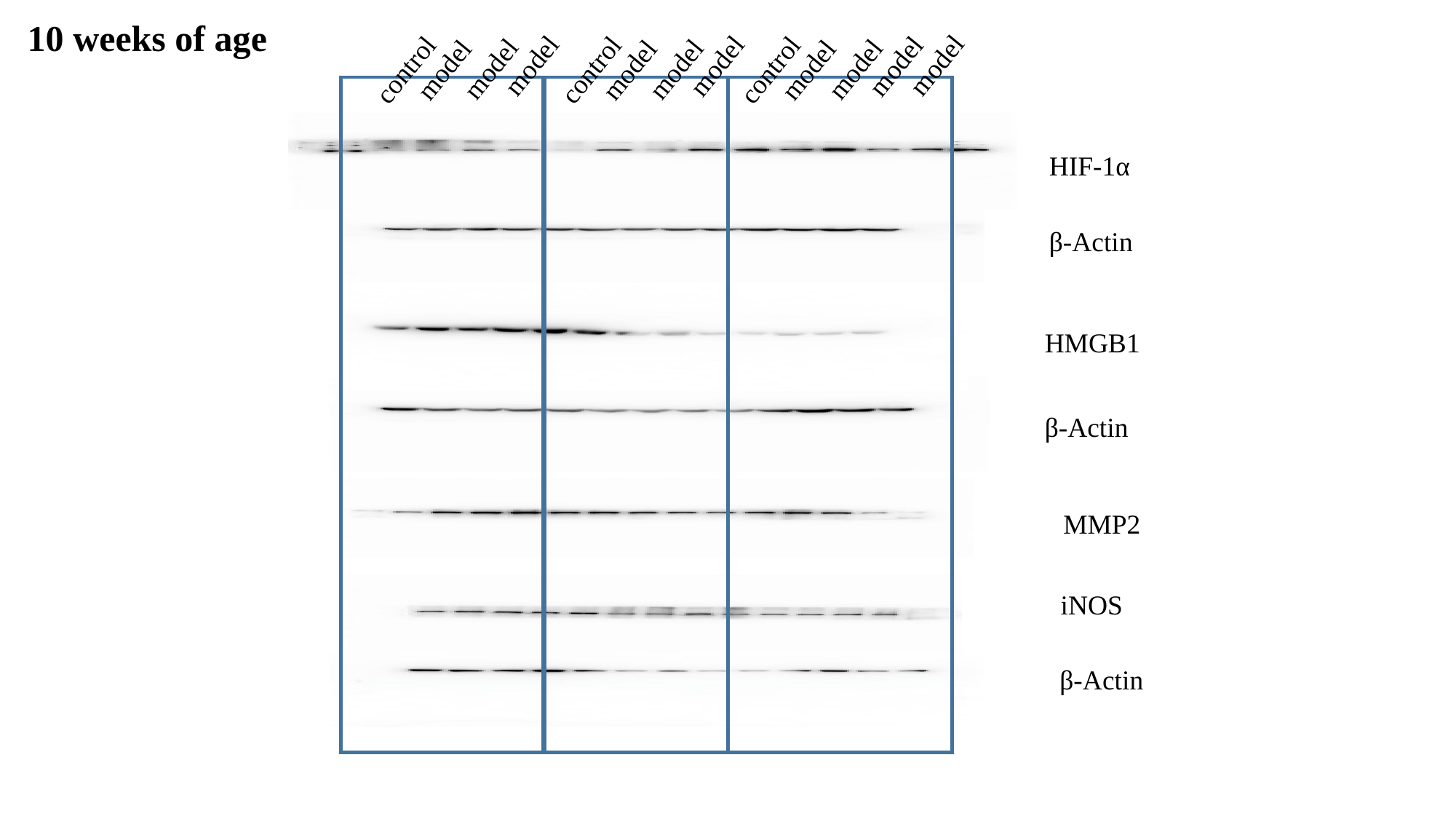

model
model
model
control
model
model
model
control
model
model
model
control
model
10 weeks of age
HIF-1α
β-Actin
HMGB1
β-Actin
MMP2
iNOS
β-Actin

## Slide 2
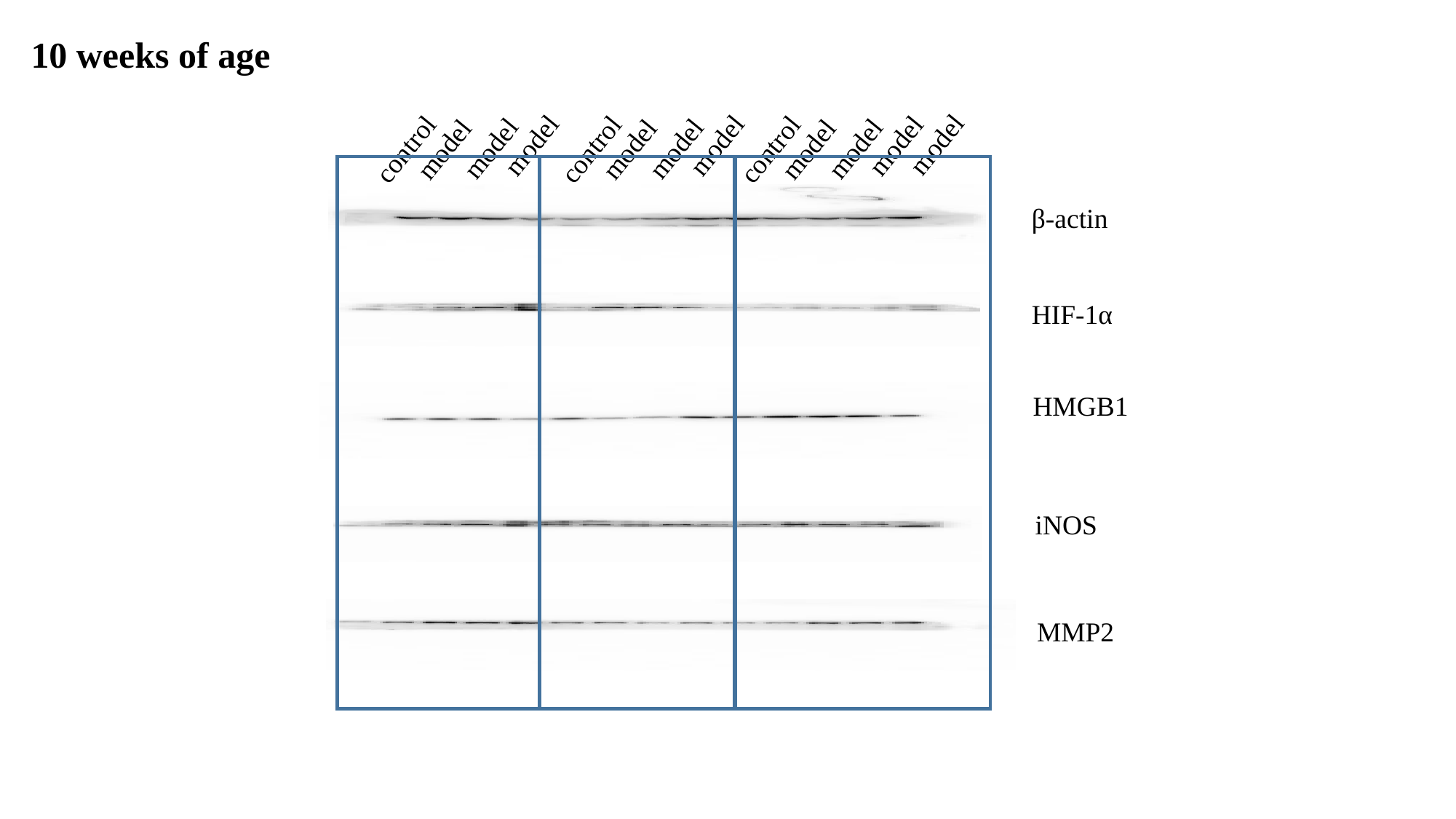

model
model
model
control
model
model
model
control
model
model
model
control
10 weeks of age
model
iNOS
β-actin
HIF-1α
HMGB1
MMP2

## Slide 3
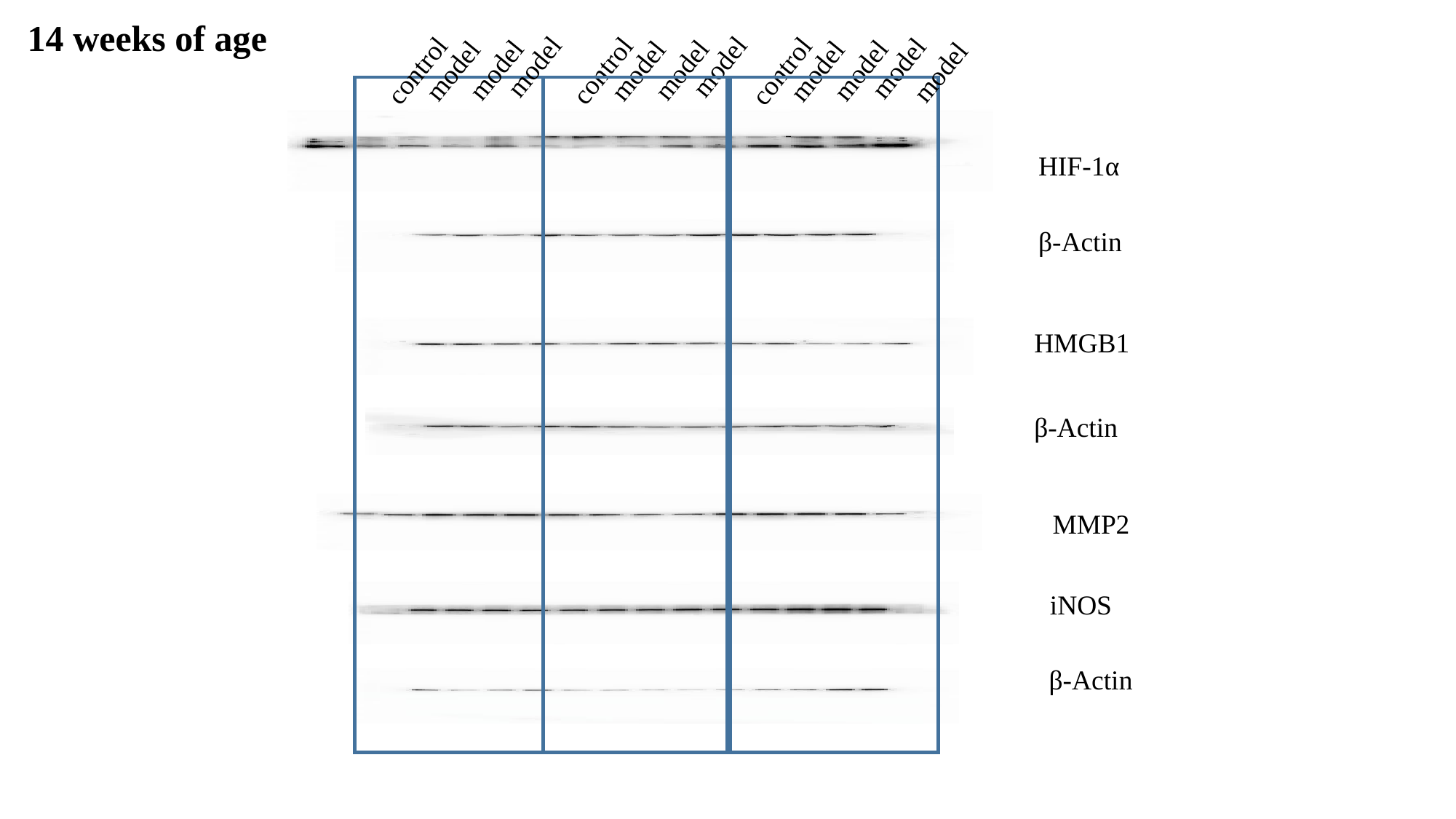

model
model
model
control
model
model
model
control
model
model
model
control
14 weeks of age
model
HIF-1α
β-Actin
HMGB1
β-Actin
MMP2
iNOS
β-Actin

## Slide 4
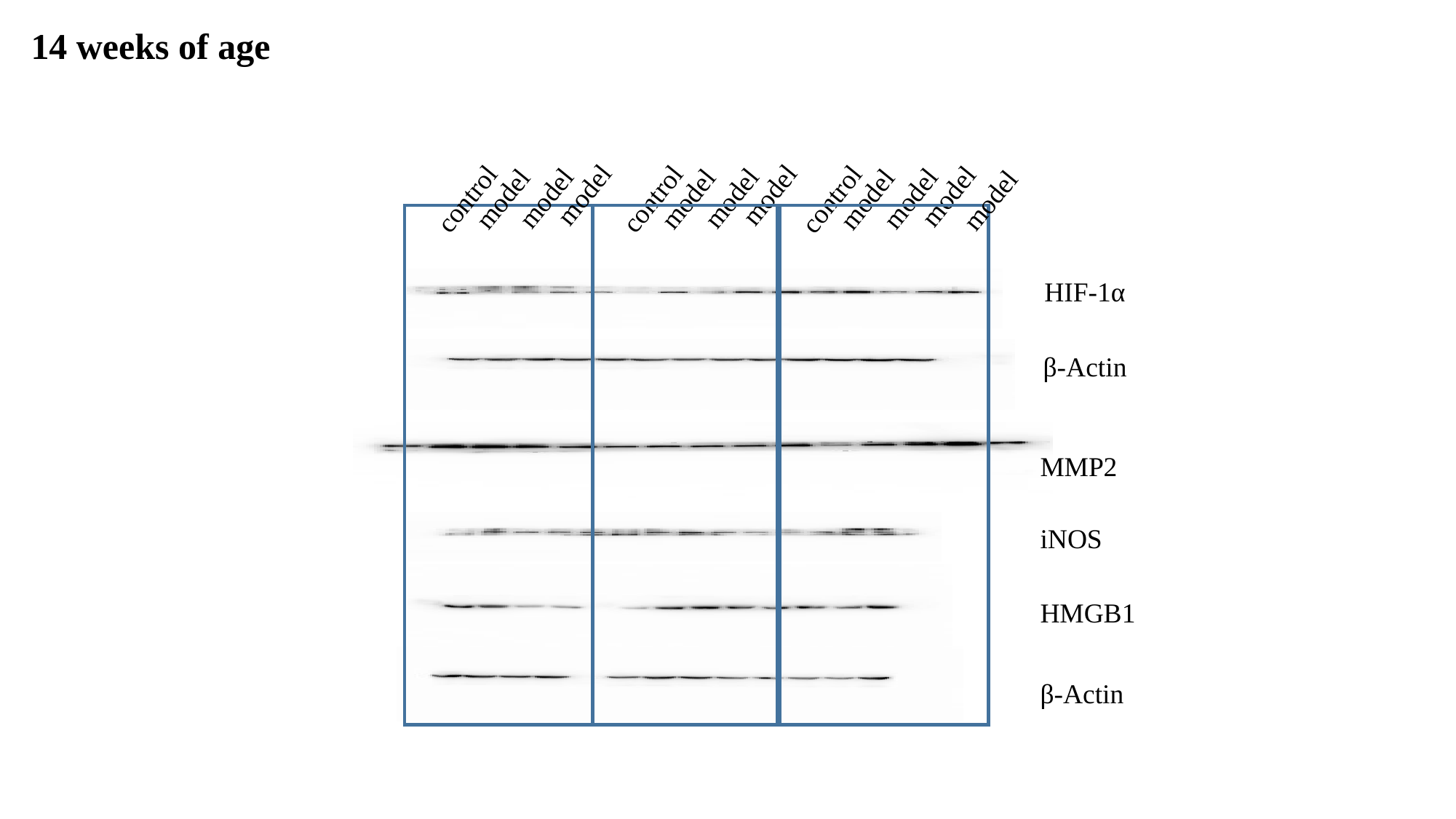

14 weeks of age
model
model
model
control
model
model
model
control
model
model
model
control
model
HIF-1α
β-Actin
iNOS
HMGB1
β-Actin
MMP2
