# Supplementary material for: Targeting Myadm to Intervene Pulmonary Hypertension on Rats Before Pregnancy Alleviates the Effect on Their Offspring’s Cardiac-Cerebral Systems
Source: Front Pharmacol. 2022 Jan 18;12:791370. doi: 10.3389/fphar.2021.791370 (PMC8804385; doi:10.3389/fphar.2021.791370)
Supplement: Supplementary file 10 [file Presentation6.PPTX]

## Slide 1
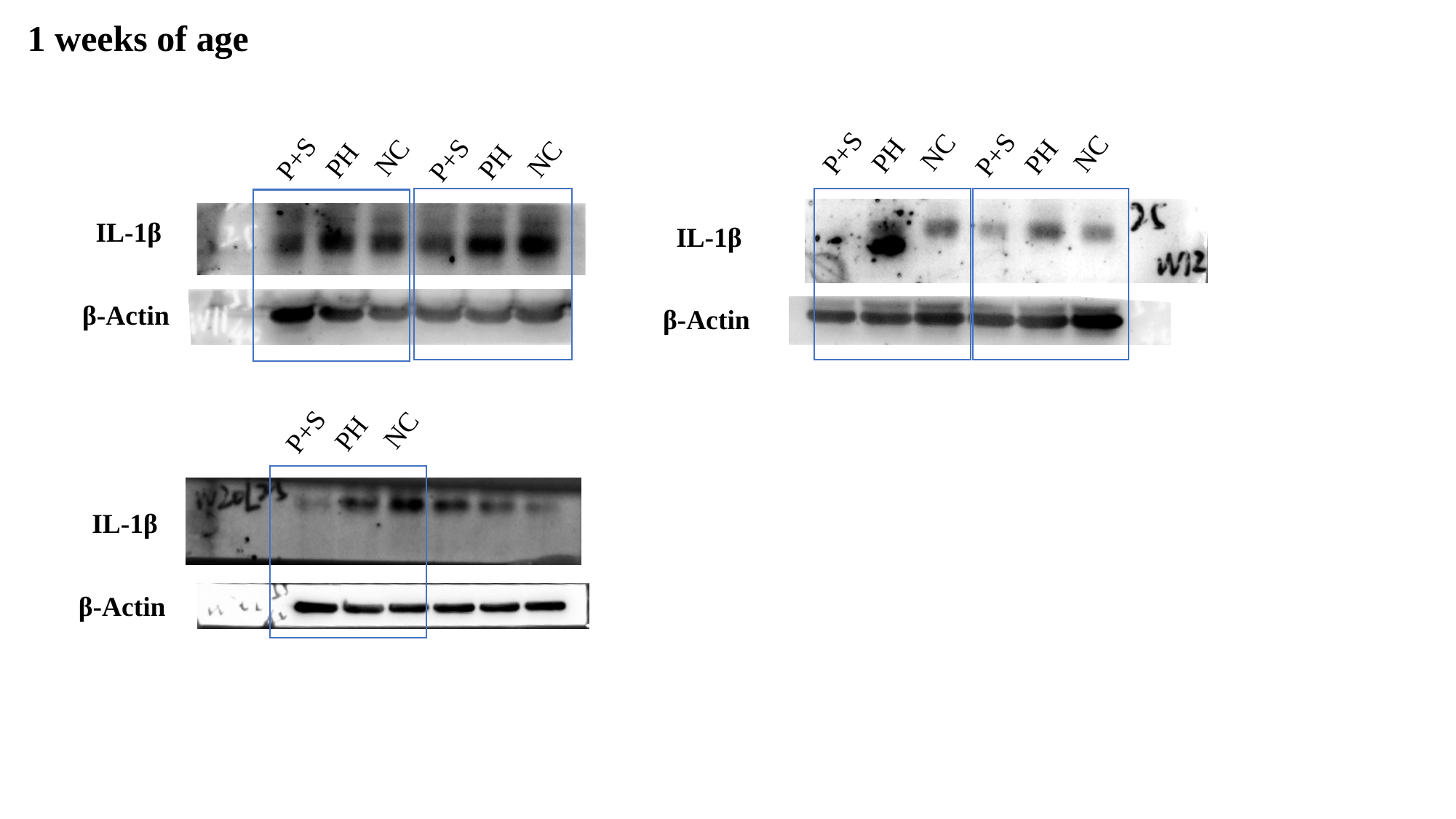

1 weeks of age
NC
NC
PH
PH
P+S
NC
P+S
NC
PH
PH
P+S
P+S
IL-1β
IL-1β
β-Actin
β-Actin
NC
PH
P+S
IL-1β
β-Actin

## Slide 2
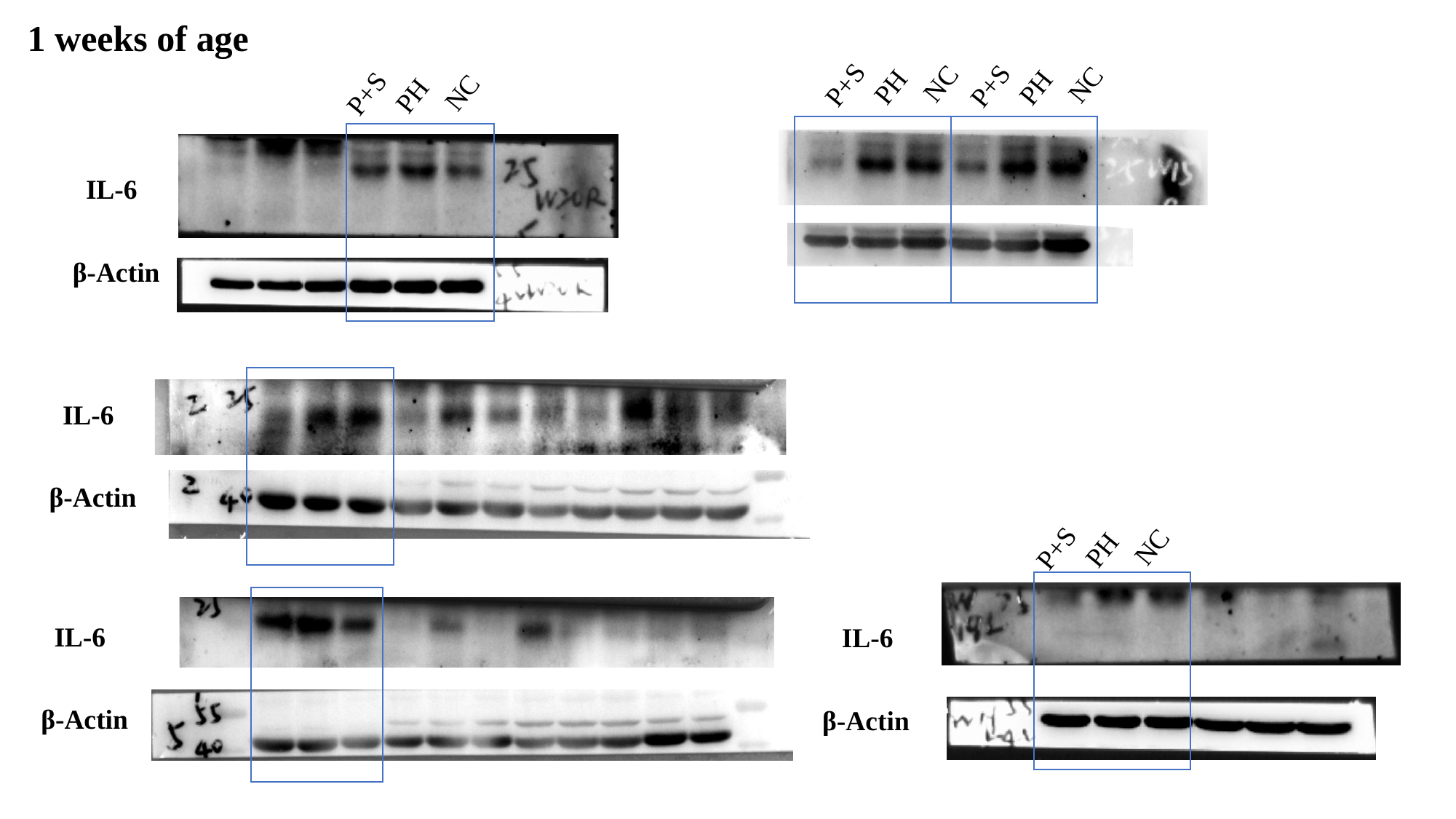

1 weeks of age
NC
NC
PH
PH
P+S
P+S
NC
PH
P+S
IL-6
β-Actin
IL-6
β-Actin
NC
PH
P+S
IL-6
IL-6
β-Actin
β-Actin

## Slide 3
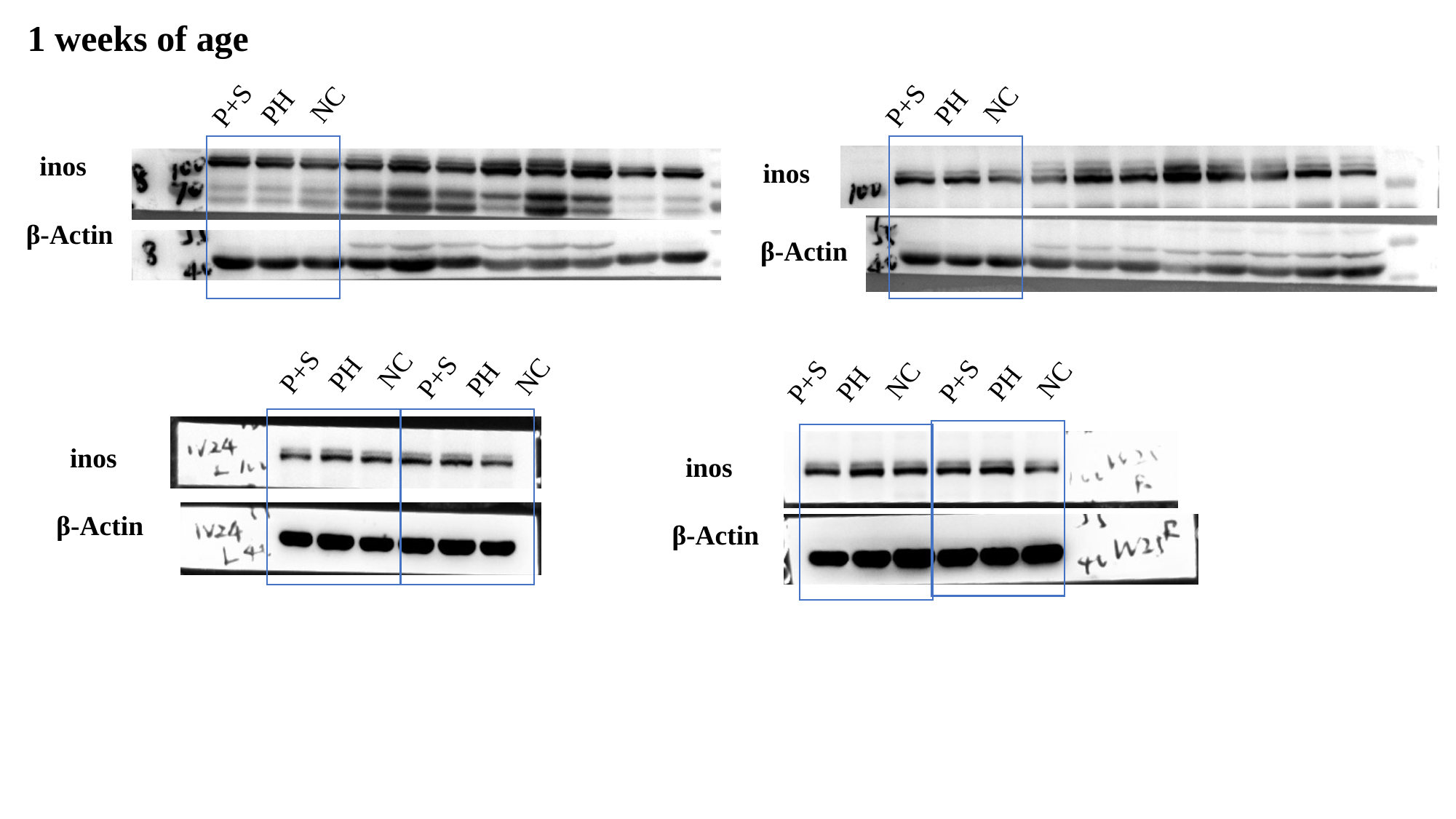

1 weeks of age
NC
NC
PH
PH
P+S
P+S
inos
inos
β-Actin
β-Actin
NC
PH
P+S
NC
PH
NC
P+S
NC
PH
PH
P+S
P+S
inos
inos
β-Actin
β-Actin

## Slide 4
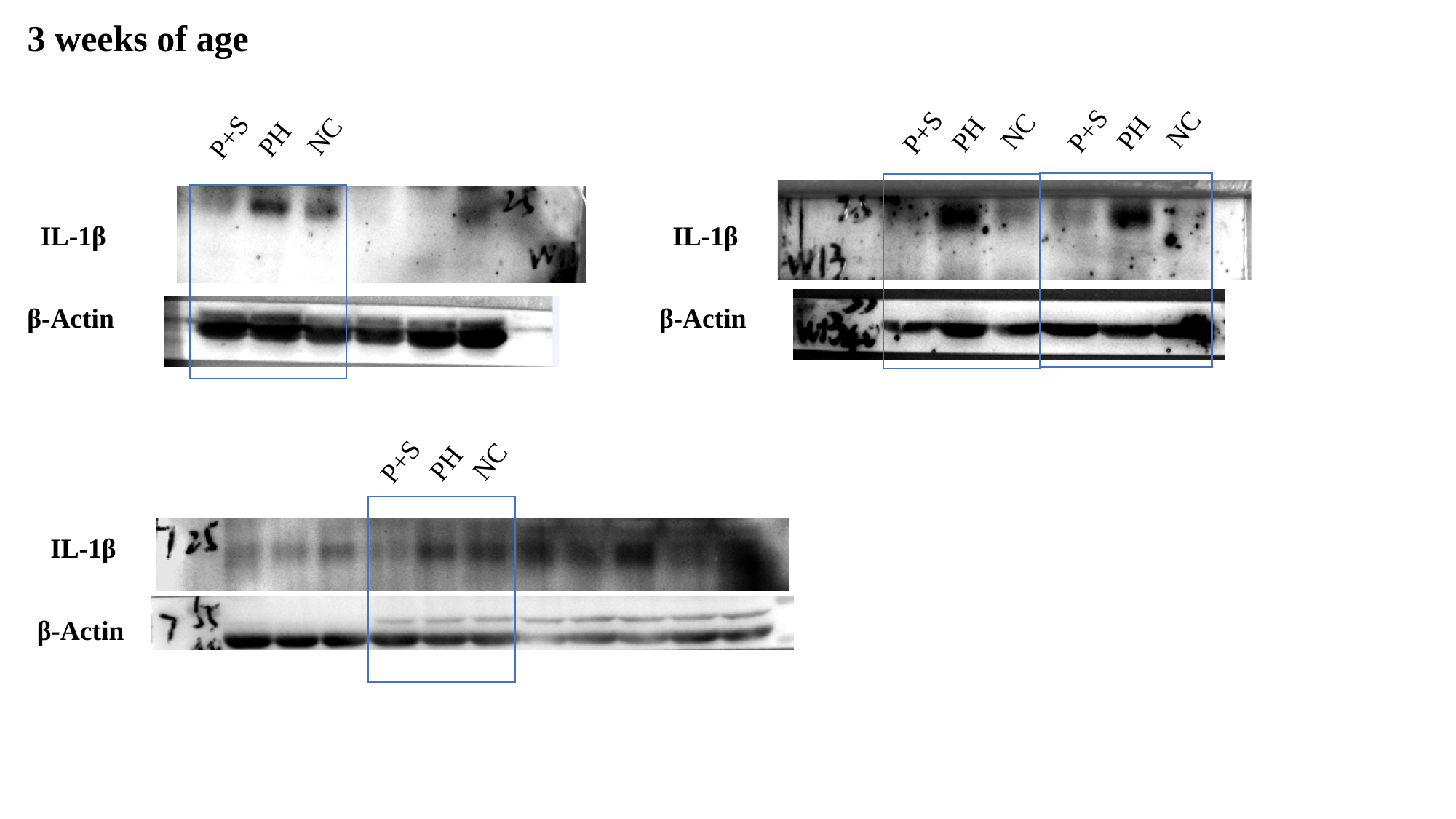

3 weeks of age
NC
NC
PH
PH
P+S
P+S
NC
PH
P+S
IL-1β
IL-1β
β-Actin
β-Actin
NC
PH
P+S
IL-1β
β-Actin

## Slide 5
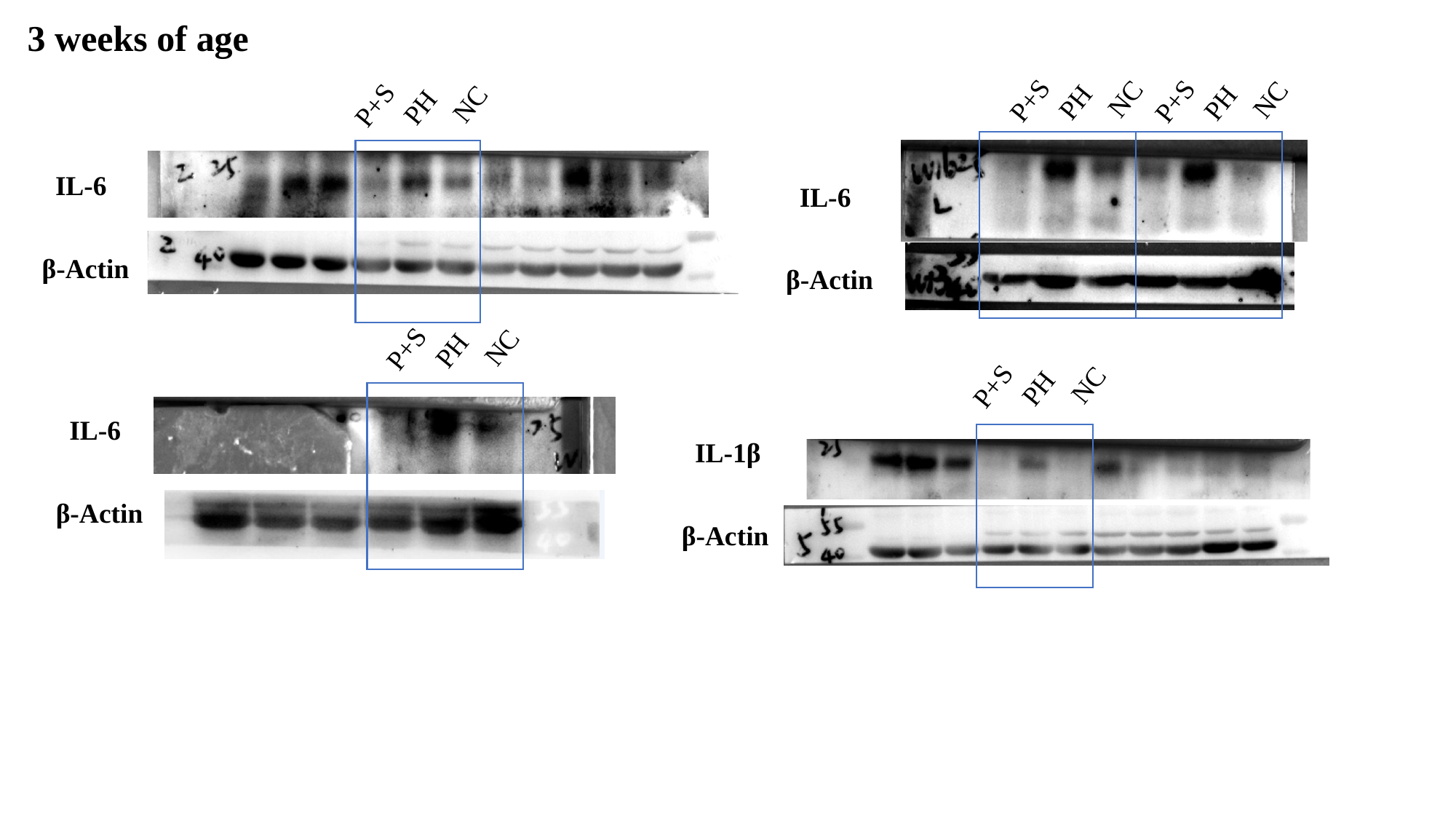

3 weeks of age
NC
NC
PH
PH
P+S
NC
P+S
PH
P+S
IL-6
IL-6
β-Actin
β-Actin
NC
PH
P+S
NC
PH
P+S
IL-6
IL-1β
β-Actin
β-Actin

## Slide 6
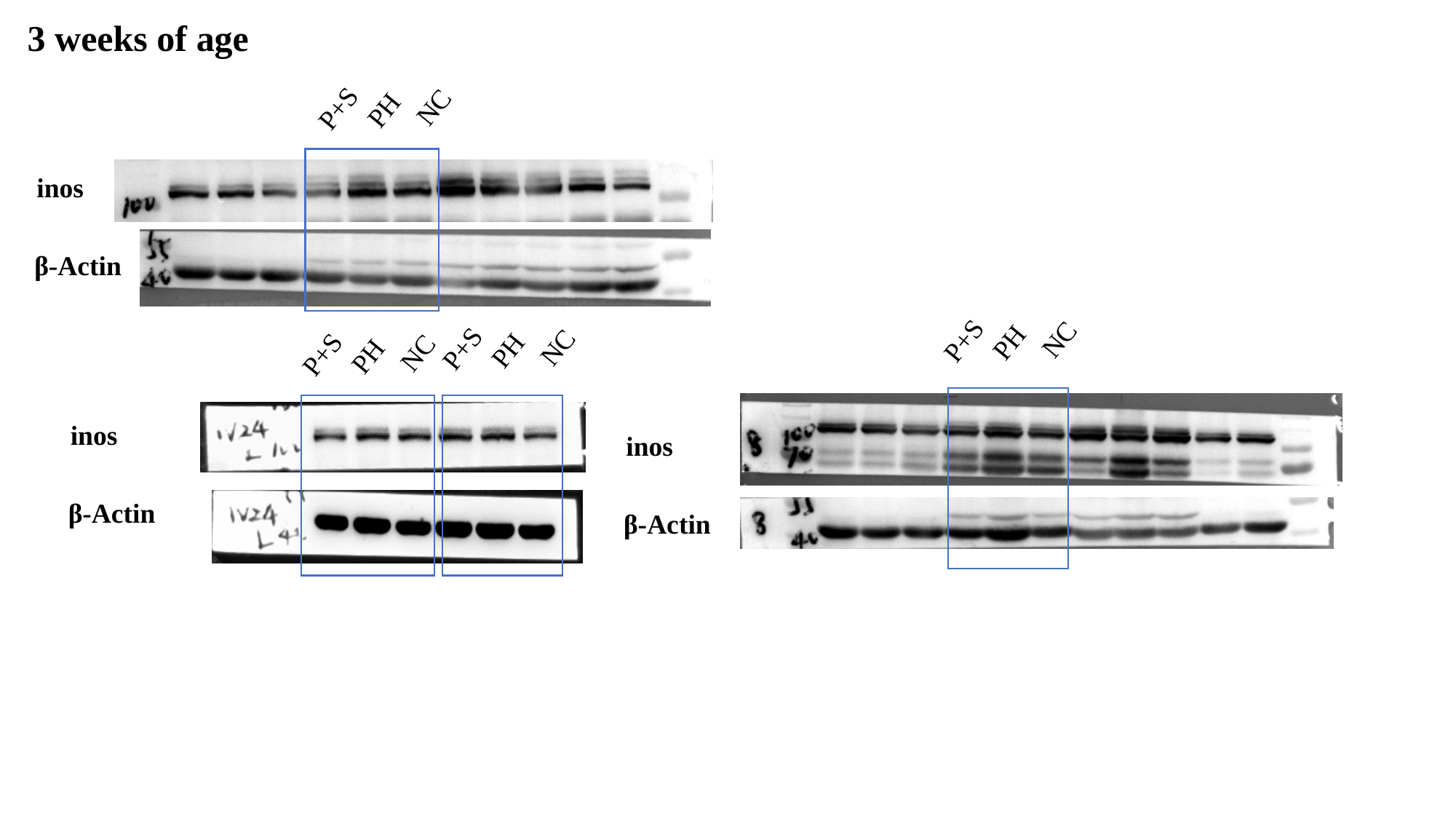

3 weeks of age
NC
PH
P+S
inos
β-Actin
NC
PH
P+S
NC
PH
P+S
NC
PH
P+S
inos
inos
β-Actin
β-Actin

## Slide 7
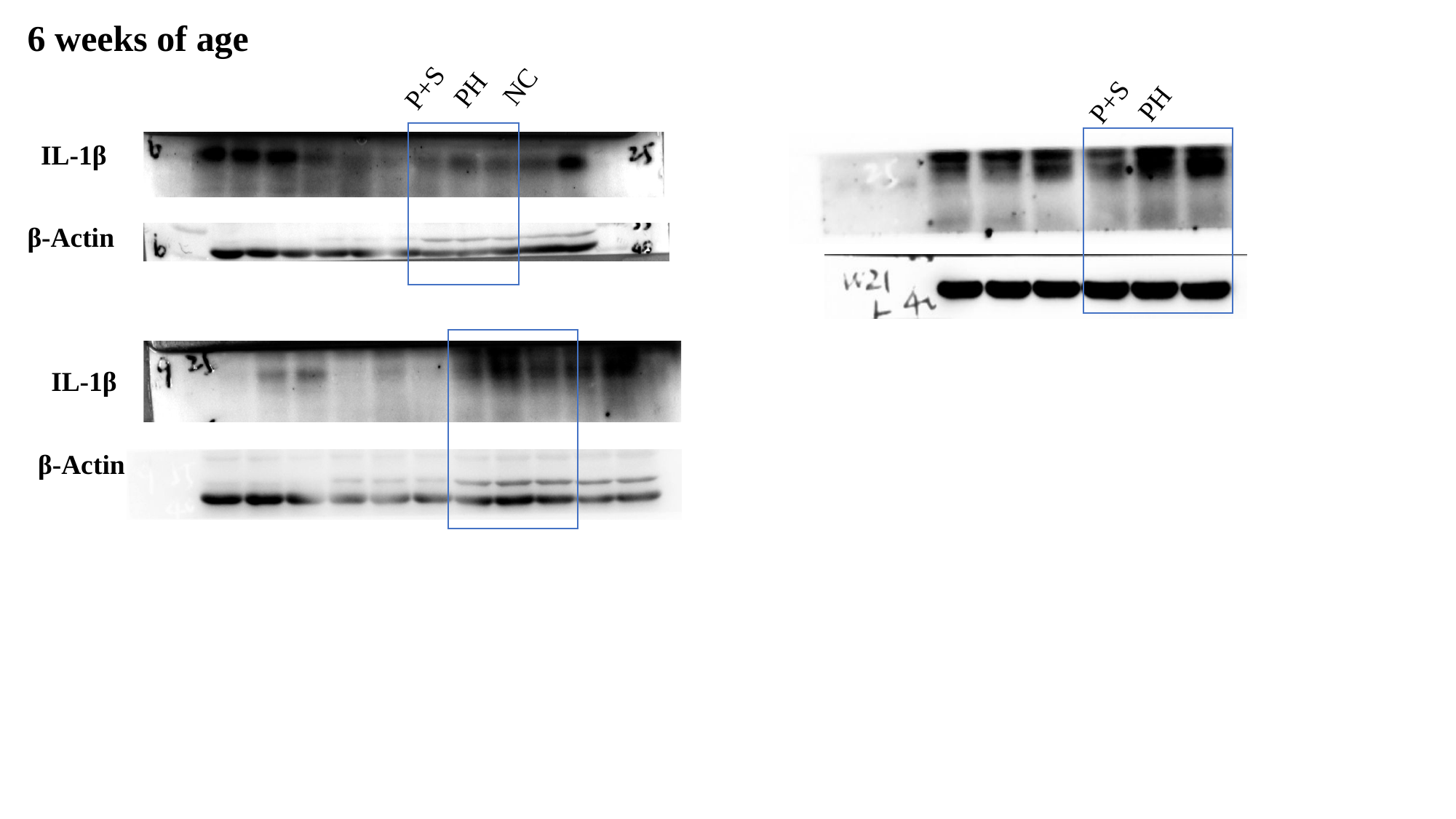

6 weeks of age
NC
PH
P+S
PH
P+S
IL-1β
β-Actin
IL-1β
β-Actin

## Slide 8
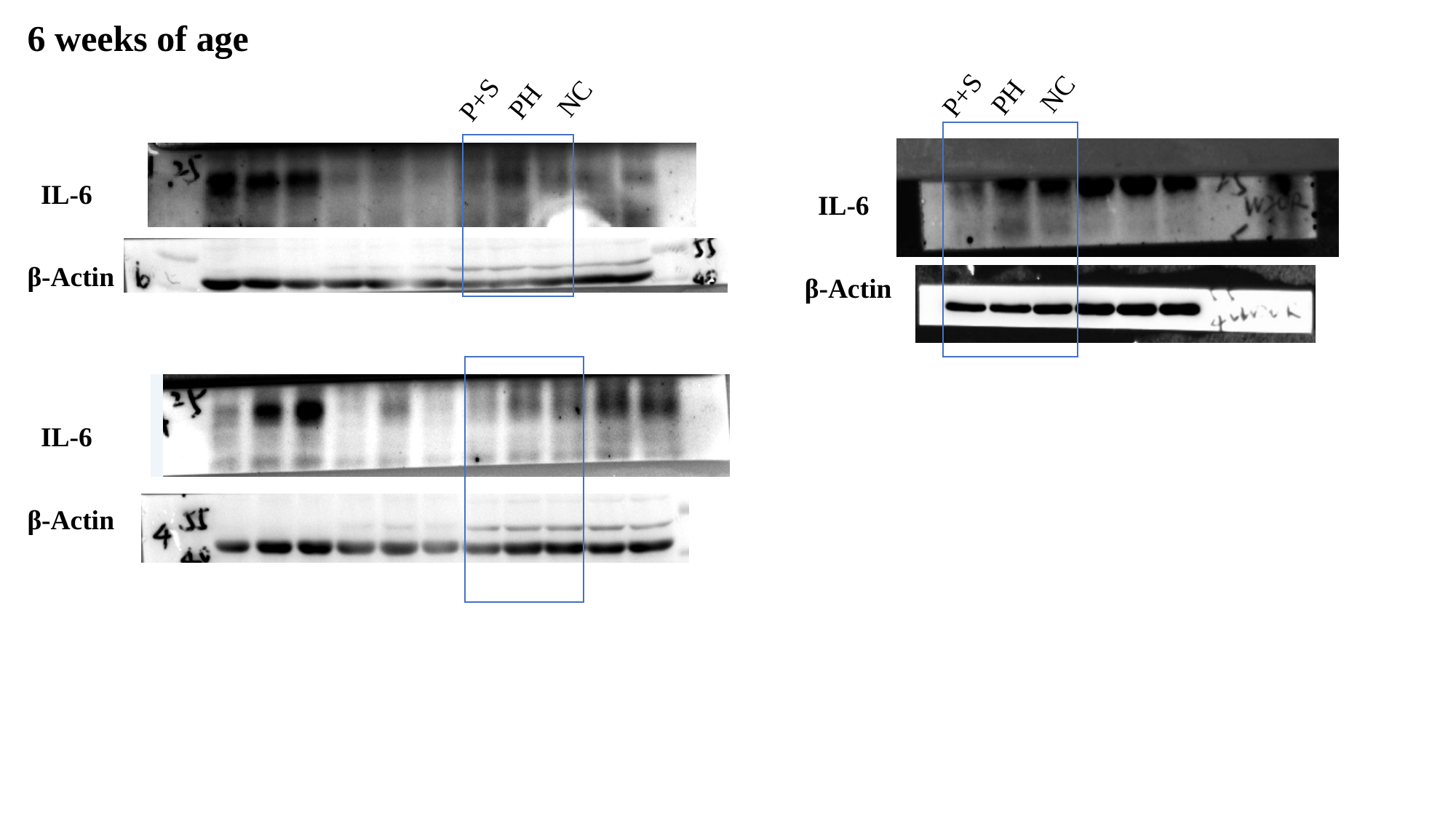

6 weeks of age
NC
PH
P+S
NC
PH
P+S
IL-6
IL-6
β-Actin
β-Actin
IL-6
β-Actin

## Slide 9
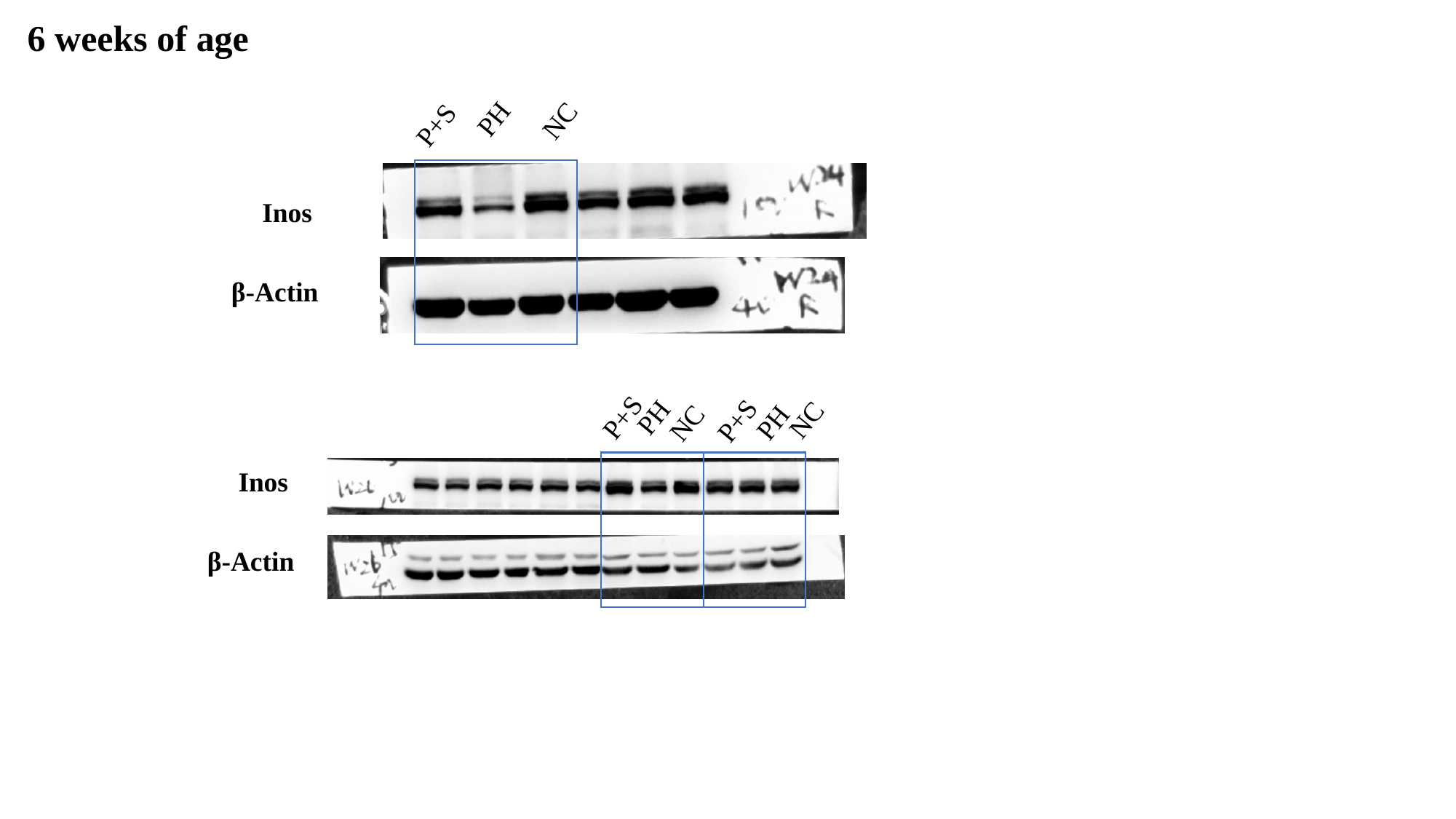

6 weeks of age
PH
NC
P+S
Inos
β-Actin
PH
NC
P+S
PH
NC
P+S
Inos
β-Actin
