# Supplementary material for: Targeting Myadm to Intervene Pulmonary Hypertension on Rats Before Pregnancy Alleviates the Effect on Their Offspring’s Cardiac-Cerebral Systems
Source: Front Pharmacol. 2022 Jan 18;12:791370. doi: 10.3389/fphar.2021.791370 (PMC8804385; doi:10.3389/fphar.2021.791370)
Supplement: Supplementary file 11 [file DataSheet1.pdf]

Supplementary Material

Supplementary Figure 1B showed the experimental setup for the establishment of pregnancy with PH rat model. After they got pregnant, their right heart function were evaluated by ultrasound. Supplementary Figure 1A showed the pulsed-wave Doppler of pulmonary flow in each group. Compared with normal pregnancy group, the spectrum showed peak reduction in pregnancy with PH group. In addition, yellow arrow indicated the second wave peak, which is one of the pulmonary flow characteristics of PH. Moreover, the maternal rats in each group were sacrificed at the end point of the study. Then, their pulmonary arteries and hearts were collected and fixed with 4% paraformaldehyde, embedded in paraffin, sliced into 5μm sections and then stained for haematoxylin and eosin (H&E) staining. Compared with normal pregnancy group, pulmonary artery of maternal PH rats were thickened, and their right ventricular cavity increased, which indicated the present of PH (Supplementary Figure 1C). Collectively, pregnancy with MCT-induced PH rat model was established successfully.

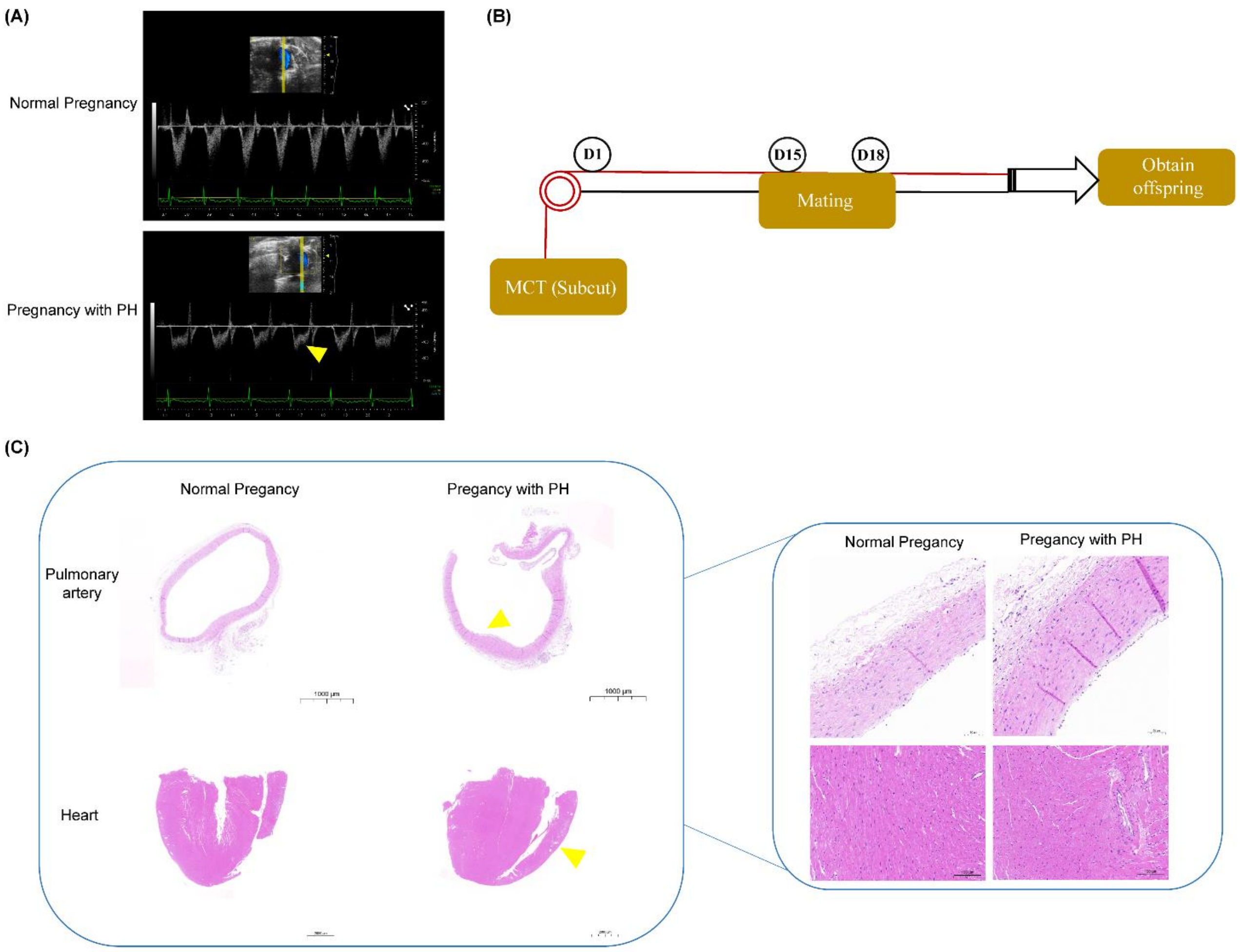

Supplementary Figure 1. The establishment and evaluation of pregnancy with MCT-induced PH rat model. A. Representative example of the pulsed-wave Doppler of pulmonary flow recorded in each group. Yellow arrow indicates the second wave peak, which is one of the pulmonary flow characteristics of PH. B. Schematic of the experimental setup for the establishment of pregnancy with MCT-induced PH rat model. C. Representative H&E staining of pulmonary artery and heart of maternal rat in each group. The left images show the entire pulmonary artery (1.2× magnification) and heart (0.6× magnification). The bar indicates 1000 μm (pulmonary artery) and 2000 μm (heart), respectively. The right images show the section of pulmonary artery (20.0× magnification) and right ventricular wall (10.0× magnification). The bar indicates 50 μm (pulmonary artery) and 100 μm (heart), respectively. Yellow arrows show thickened pulmonary artery and increased right ventricular cavity. PH, pulmonary hypertension.

Supplementary Figure 2B showed the experimental setup for the establishment of shRNA-Myadm intervene maternal PH rat model. And the interference efficiency of shRNA-Myadm evaluated by western blot analysis and RT-qPCR (Supplementary Figure 2C). After they got pregnant, their right heart function were evaluated by ultrasound. Supplementary Figure 2A showed the pulsed-wave Doppler of pulmonary flow in each group. Compared with normal control, the spectrum showed decreased peak in PH group. In addition, yellow arrow indicated the second wave peak, which is one of the pulmonary flow characteristics of PH. However, silencing Myadm of PH rats could alleviate their pulmonary flow. Moreover, the maternal rats in each group were sacrificed at the end point of the study. Then, their pulmonary arteries and hearts were collected and fixed with 4% paraformaldehyde, embedded in paraffin, sliced into 5μm sections and then stained for haematoxylin and eosin (H&E) staining. Compared with normal control, pulmonary artery of PH rats were thickened, and their right ventricular cavity increased, which could be alleviated by Myadm intervention (Supplementary Figure 2D), which consisted with our previous findings.

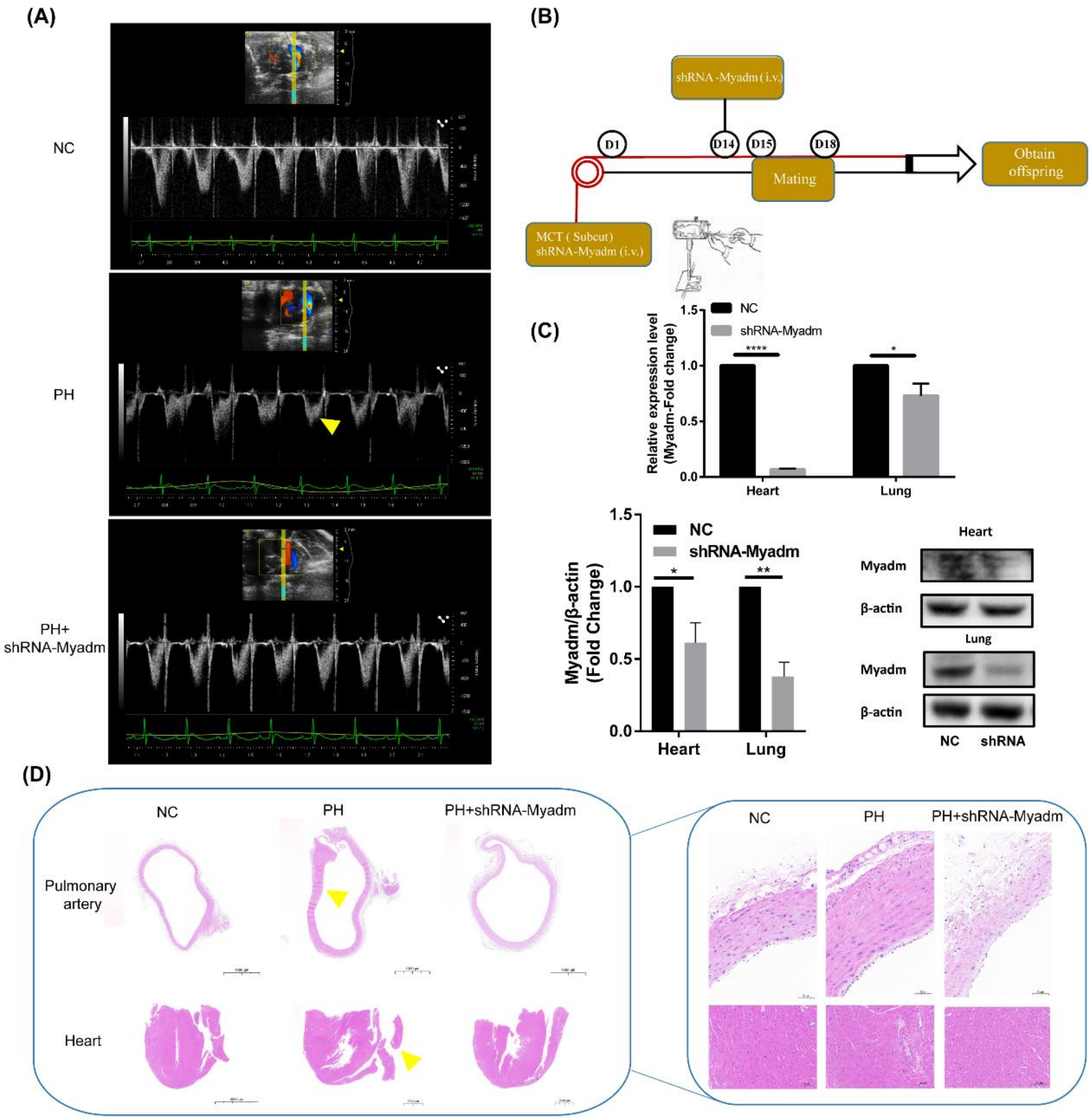

Supplementary Figure 2. The establishment and evaluation of shRNA-Myadm intervene maternal PH rat model. A. Representative example of the pulsed-wave Doppler of pulmonary flow recorded in each group. Yellow arrow indicates the second wave peak, which is one of the pulmonary flow characteristics of PH. B. Schematic of the experimental setup for the establishment of shRNA-Myadm intervene maternal PH rat model. C. The interference efficiency of shRNA-Myadm evaluated by western blot analysis and RT-qPCR (n=3 rats per group). D. Representative H&E staining of pulmonary artery and heart of maternal rat in each group. The left images show the entire pulmonary artery (1.4× magnification) and heart (0.4× magnification). The bars indicate 1000 μm (pulmonary artery) and 2000 μm (heart), respectively. The right images show the section of pulmonary artery (20.0× magnification) and right ventricular wall (10.0× magnification). The bars both indicate 50 μm. Yellow arrows show thickened pulmonary artery and increased right ventricular cavity. NC, normal control. PH, pulmonary hypertension.

In the main text, we explored the long-term effects of maternal PH on the whole rat offspring for the first time. Half male and half female offspring were contained in every examination. In supplementary materials, the data of male and female offspring subgroups are placed with the results of whole group offspring in the following four figures.

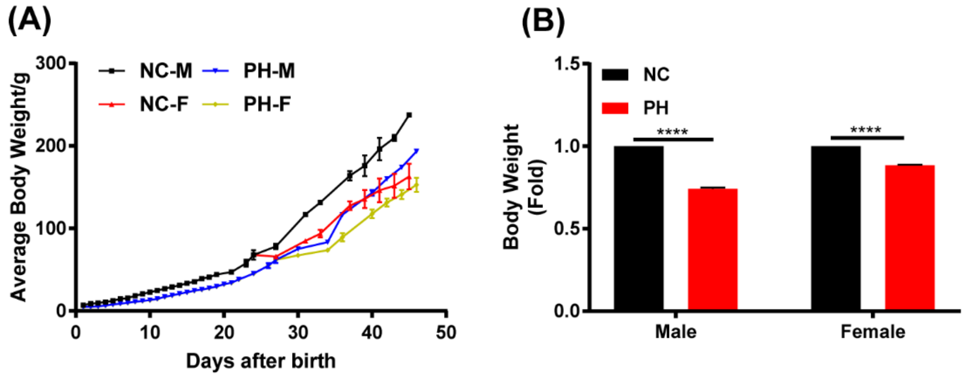

Supplementary Figure 3. The growth of offspring in different genders and groups. A. The growth of average body weight in different genders and groups (mean±standard deviation). B. The fold change of body weight of maternal PH offspring in the same gender compared with normal offspring at seven weeks of age. NC, normal pregnancy group; PH, pregnancy with PH group; M, male; F, female. ****p<0.0001 vs the normal pregnancy group (n=3 rats per group).

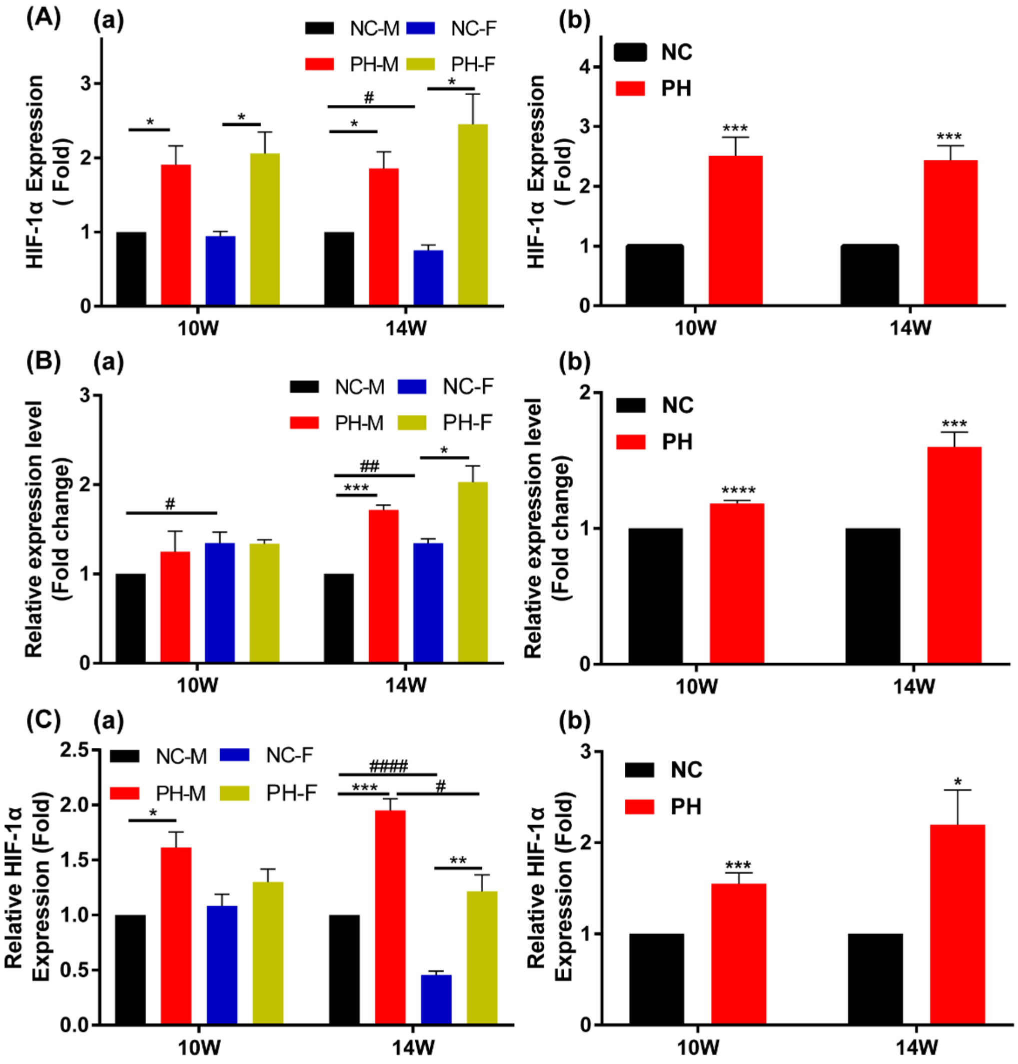

Supplementary Figure 4. Cerebral hypoxia existed in the offspring of the PH group. A. Detected by ELISA. B. Detected by RT-qPCR; C. Detected by Western blot analysis. (a). Each result with sex subgroup (n=3 rats per group); (b). Each result of the whole group of offspring (n=6 rats per group with half male and half female). NC, normal pregnancy group; PH, pregnancy with PH group; M, male; F, female. * represents the statistical significance between NC and PH. # represents the statistical significance between male and female rats in the same group. *(#)p<0.05, **(##) p<0.01, ***p<0.001, ****(####)p<0.0001

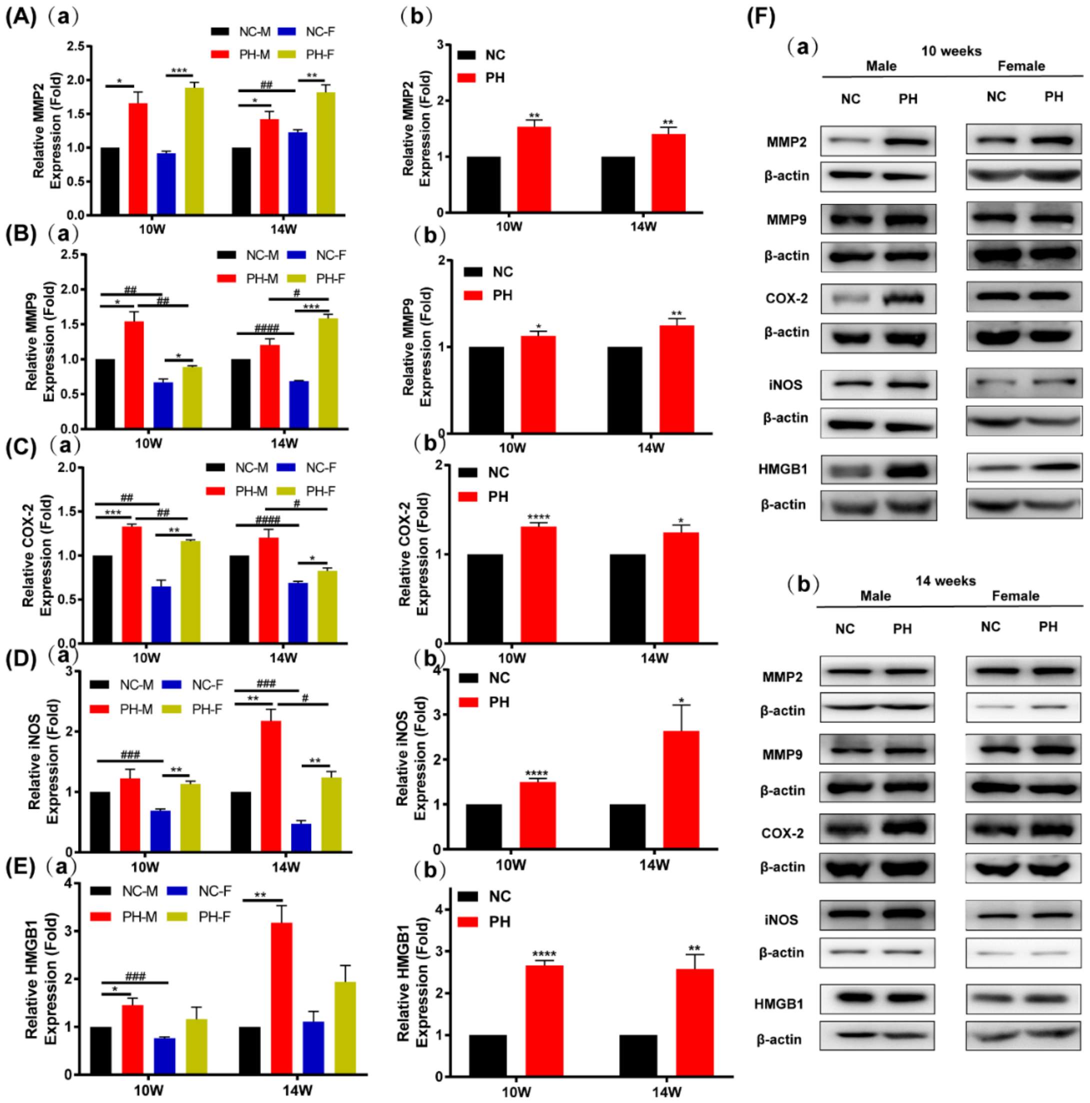

Supplementary Figure 5. Cerebral inflammation existed in the offspring of the PH pregnancy group. A. Summary of MMP2 expression in the cerebral cortex of offspring in each group at different ages. B. Summary of MMP9 expression in the cerebral cortex of offspring in each group at different ages. C. Summary of COX-2 in the cerebral cortex of offspring in each group at different ages. D. Summary of iNOS in the cerebral cortex of offspring in each group at different ages. E. Summary of HMGB1 in the cerebral cortex of offspring in each group at different ages. (a). Each result with sex subgroup (n=3 rats per group); (b). Each result of the whole group of offspring (n=6 rats per group with half male and half female). F. Western blot analysis of MMP2, MMP9, COX-2, iNOS and HMGB1 in the cerebral cortex of offspring in each group at 10 weeks of age (a) and 14 weeks of age (b). NC, normal pregnancy group; PH, pregnancy with PH group; M, male; F, female. * represents the statistical significance between NC and PH. # represents the statistical significance between male and female rats in the same group. *(#)p<0.05, **(##)p<0.01, ***(###)p<0.001, ****(####) p<0.0001

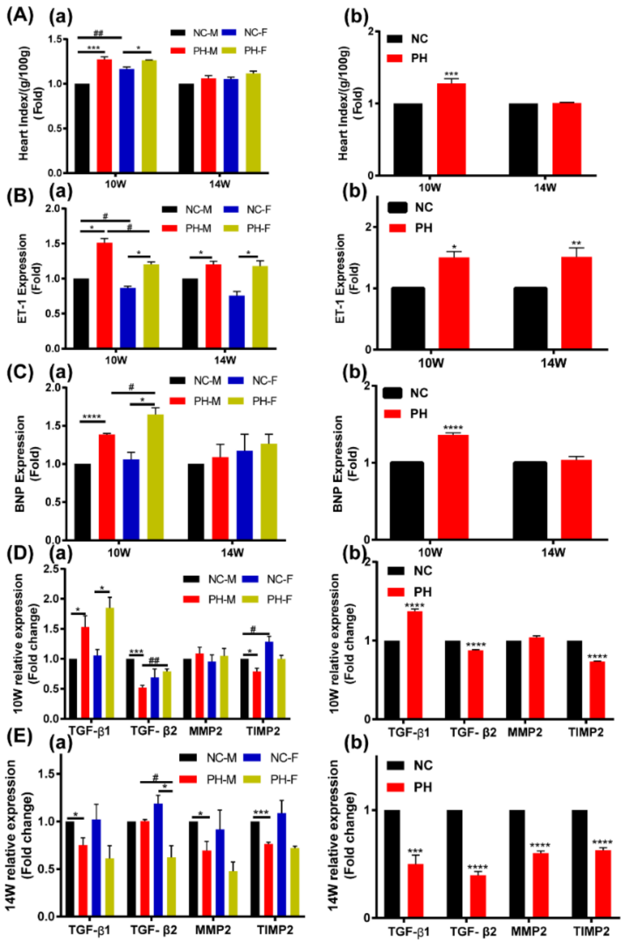

Supplementary Figure 6. Abnormal cardiac changes in offspring in the PH pregnancy group. A. The heart index (weight of heart/body weight) of offspring at ten weeks of age and fourteen weeks of age in each group. B. The expression change of ET-1 in the plasma in each group at ten weeks of age and fourteen weeks of age determined by ELISA. C. The expression change of BNP in the heart tissue in each group at ten weeks of age and fourteen weeks of age determined by ELISA. D. The mRNA expression of TGF-β1, TGF-β2, MMP2 and TIMP2 in the heart tissue of offspring in each group at ten weeks of age. E. The mRNA expression of TGF-β1, TGF-β2, MMP2 and TIMP2 in the heart tissue of offspring in each group at fourteen weeks of age. (a). Each result with sex subgroup (n=3 rats per group); (b). Each result of the whole group of offspring (n=6 rats per group with half male and half female). NC, normal pregnancy group; PH, pregnancy with PH group; M, male; F, female. * represents the statistical significance between NC and PH. # represents the statistical significance between male and female rats in the same group. *(#)p<0.05, **(##)p<0.01, ***p<0.001, **** p<0.0001

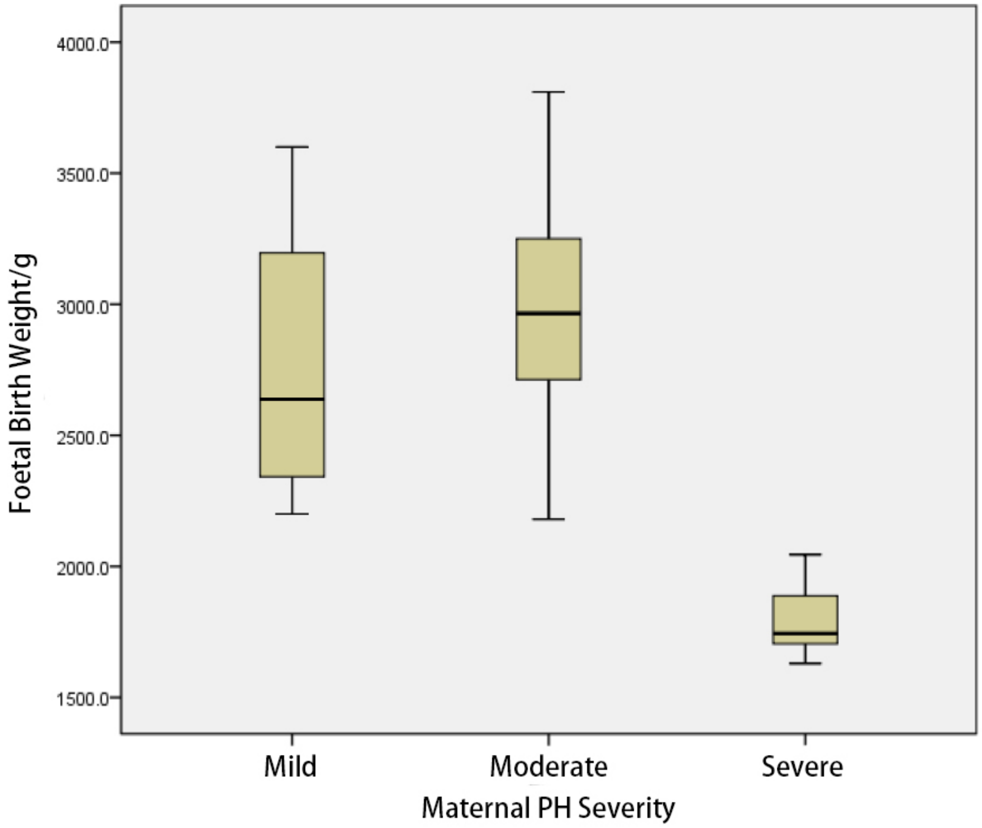

Supplementary Figure 7. Boxplot of maternal PH severity and foetal birth weight.

Supplementary Table 1 Normal distribution text of involved cases

	**Maternal PH severity**	**Kolmogorov-Smirnova**			**Shapiro-Wilk**		
		Statistics	df	Sig.	Statistics	df	Sig.
**Foetal birth weight**	Mild	0.235	4	.	0.937	4	0.636
	Moderate	0.128	14	0.200*	0.962	14	0.757
	Severe	0.253	8	0.142	0.909	8	0.344

PH, pulmonary hypertension; * This is the lower limit of the true significance level.

Supplementary Table 2 Test for homogeneity of variance of involved cases

**Foetal birth weight**			
Levene statistics	df1	df2	Sig.
2.774	2	23	0.083

Supplementary Table 3 Correlations between maternal age and foetal birth weight

**Item**	**Index**	**Maternal age**	**Foetal birth weight**
**Maternal age**	Pearson Correlation	1	0.258
	Sig.(2-tail)		0.203
**Foetal birth weight**	Pearson Correlation	0.258	1
	Sig.(2-tail)	0.203	

**. Correlation is significant at the 0.01 level (2-tailed).

Supplementary Table 4 Maternal weights and pulmonary artery pressure of rat model in each group

**Group**	**Maternal weights/g**	**Maternal pulmonary artery pressure/mmHg (Systolic / diastolic blood pressure)**
Normal Pregnancy	280	27/15
	260	22/12
PH	240	36/13
	240	38/24
Pregnancy with PH	265	39/13
	246	37/24
