# Supplementary material for: Targeting Myadm to Intervene Pulmonary Hypertension on Rats Before Pregnancy Alleviates the Effect on Their Offspring’s Cardiac-Cerebral Systems
Source: Front Pharmacol. 2022 Jan 18;12:791370. doi: 10.3389/fphar.2021.791370 (PMC8804385; doi:10.3389/fphar.2021.791370)
Supplement: Supplementary file 13 [file Presentation3.PPTX]

## Slide 1
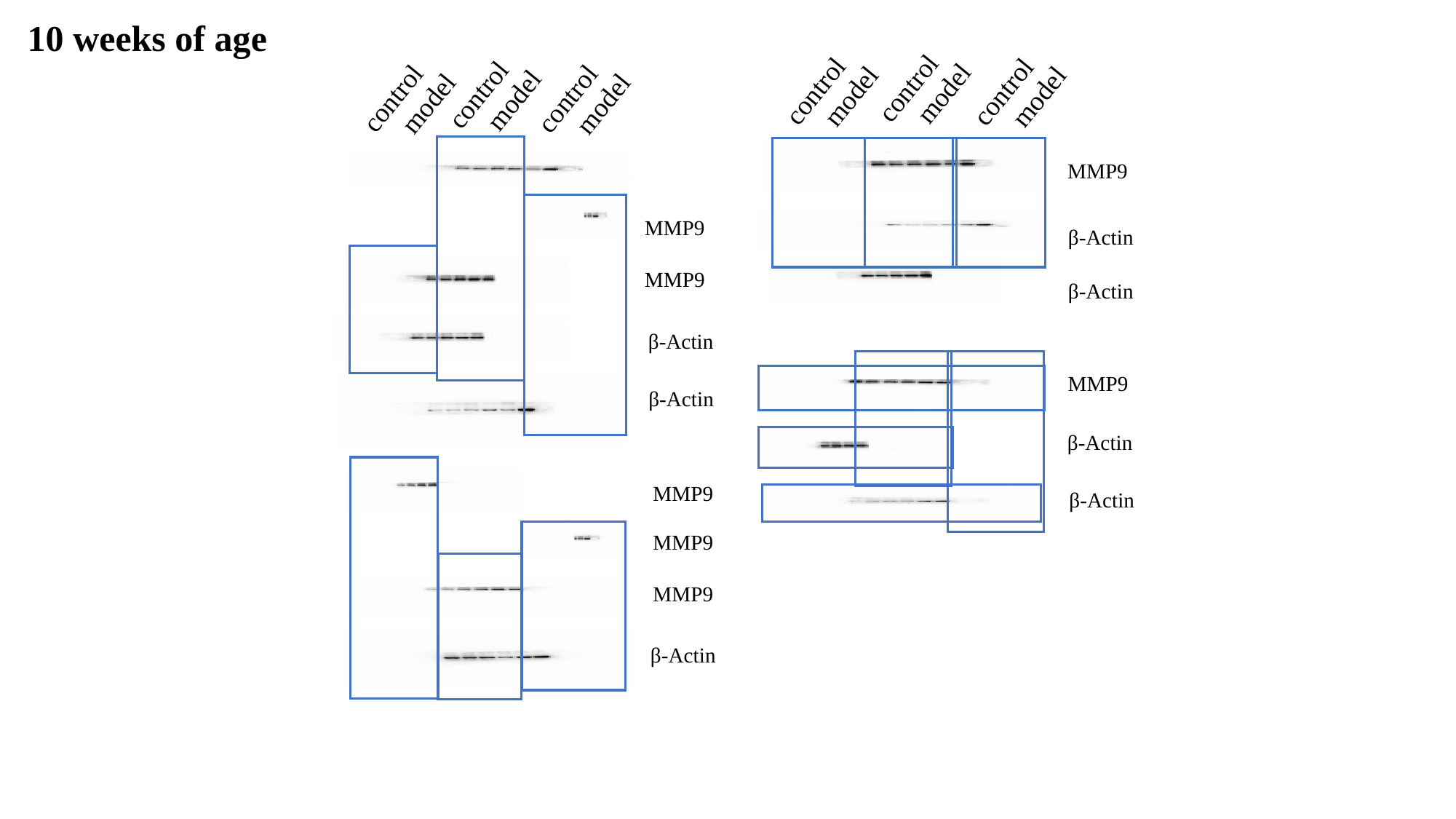

control
model
control
model
control
model
control
model
control
model
control
model
10 weeks of age
MMP9
MMP9
β-Actin
β-Actin
MMP9
β-Actin
β-Actin
MMP9
β-Actin
MMP9
β-Actin
MMP9
MMP9
β-Actin
